# Digital Light 3D Printed Bioresorbable and NIR‐Responsive Devices with Photothermal and Shape‐Memory Functions

**DOI:** 10.1002/advs.202200907

**Published:** 2022-07-27

**Authors:** Nevena Paunović, Jessica Marbach, Yinyin Bao, Valentine Berger, Karina Klein, Sarah Schleich, Fergal Brian Coulter, Nicole Kleger, André R. Studart, Daniel Franzen, Zhi Luo, Jean‐Christophe Leroux

**Affiliations:** ^1^ Institute of Pharmaceutical Sciences Department of Chemistry and Applied Biosciences ETH Zurich Zurich 8093 Switzerland; ^2^ Musculoskeletal Research Unit Vetsuisse Faculty University of Zurich Zurich 8057 Switzerland; ^3^ Complex Materials Department of Materials ETH Zurich Zurich 8093 Switzerland; ^4^ Department of Pulmonology University Hospital Zurich Zurich 8006 Switzerland; ^5^ Guangdong Provincial Key Laboratory of Advanced Biomaterials, Department of Biomedical Engineering Southern University of Science and Technology Shenzhen 518055 P. R. China

**Keywords:** 3D printing, 4D printing, gold nanorods, photothermal therapy, shape‐memory

## Abstract

Digital light processing (DLP) 3D printing is a promising technique for the rapid manufacturing of customized medical devices with high precision. To be successfully translated to a clinical setting, challenges in the development of suitable photopolymerizable materials have yet to be overcome. Besides biocompatibility, it is often desirable for the printed devices to be biodegradable, elastic, and with a therapeutic function. Here, a multifunctional DLP printed material system based on the composite of gold nanorods and polyester copolymer is reported. The material demonstrates robust near‐infrared (NIR) responsiveness, allowing rapid and stable photothermal effect leading to the time‐dependent cell death. NIR light‐triggerable shape transformation is demonstrated, resulting in a facilitated insertion and expansion of DLP printed stent ex vivo. The proposed strategy opens a promising avenue for the design of multifunctional therapeutic devices based on nanoparticle–polymer composites.

## Graphical abstract

Digital light 3D printing of biodegradable near‐infrared light‐responsive elastomeric devices with photothermal and shape‐memory functions based on biodegradable polymer and gold nanorods composites is presented.

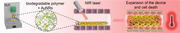

## 1 Introduction

Over the last decade, 3D printing^[^ ^1^, ^2^, ^3^ ^]^ has evolved into a promising method for the manufacturing of on‐demand personalized medical devices,^[^ ^4^, ^5^ ^]^ including prosthetics,^[^ ^6^ ^]^ mouthguards,^[^ ^7^ ^]^ stents,^[^ ^8^, ^9^ ^]^ and tracheal splints.^[^ ^10^, ^11^ ^]^ Among various 3D printing techniques, digital light processing (DLP) is particularly attractive because it enables affordable high‐speed high‐resolution manufacturing of complex‐shaped objects with smooth surfaces.^[^ ^1^, ^12^, ^13^ ^]^ One of the key advantages of DLP lies in the versatility of printable resins, which allows tuning the mechanical properties from tough and rigid to elastomers resembling soft tissues, as well as the incorporation of stimuli responsiveness to enable 4D printing.^[^ ^14^, ^15^, ^16^, ^17^ ^]^ Recently, biodegradable medical devices manufactured by DLP have been reported,^[^ ^8^, ^18^ ^]^ thus eliminating the need for additional interventions to extract them from the body.^[^ ^19^, ^20^, ^21^, ^22^ ^]^ With the increasing range of materials printable by DLP, the technology is now mature to fulfill the growing demand for 3D printed personalized devices with therapeutic functions. Several studies have reported the printing of drug‐eluting devices with controllable release kinetics. However, the mechanical characterization of these composites was generally lacking.^[^ ^23^, ^24^, ^25^ ^]^ In the case of drug‐eluting nonvascular stents, which are investigated for the local treatment of tumors in tubular organs (e.g., airways or esophagus) both the mechanical and therapeutic performances are indeed important. They should possess good mechanical resilience to open the obstructed anatomical structures while locally releasing antiproliferative drugs.^[^ ^26^, ^27^, ^28^ ^]^ Despite their promising prospects, such stents have so far failed to demonstrate superiority over the bare stents in clinical trials.^[^ ^29^, ^30^ ^]^

Besides the controlled release of drugs, light‐based approaches have also been explored for the local treatment of tumors in tubular organs.^[^ ^31^, ^32^ ^]^ Indeed, photothermal therapy (PTT) with metal nanoparticles is attractive for the local treatment of cancer due to its high efficiency and good safety profile. It relies on the thermal ablation of the tumor by protein denaturation and oxidative stress upon light irradiation of nanoparticles, while the surrounding tissue remains unaffected.^[^ ^33^, ^34^, ^35^, ^36^, ^37^ ^]^ To achieve this, the temperature of the tumor should be kept in a clinically relevant range of 41–48 °C, while the heating duration should be adapted to a specific medical application.^[^ ^37^ ^]^ Near‐infrared (NIR) light is an ideal trigger for heat‐mediated cell death thanks to its high penetration depth. Furthermore, its combination with spatiotemporally controllable application minimizes the risk of off‐target heating.^[^ ^33^, ^38^ ^]^ Gold nanorods (AuNRs) are one of the most commonly used photothermal agents, due to their biocompatibility and ability to shift their longitudinal surface plasmon resonance (SPR) to the NIR region by tuning the aspect ratio.^[^ ^33^, ^36^, ^39^, ^40^, ^41^ ^]^ When mixed with thermally‐induced shape‐memory polymers, these nanoparticles offer the opportunity for the 4D printing of “smart” devices that could be easily deployed and later expanded by NIR light.^[^ ^17^, ^42^, ^43^, ^44^, ^45^, ^46^ ^]^ Shape‐memory devices can be deformed above their transition temperature (e.g., glass transition temperature or melting point) to achieve a new shape that is then preserved by cooling the object below the transition temperature, which is referred to as the programming step. When the object is heated again above its transition point, it recovers to its original 3D printed shape.^[^ ^47^, ^48^, ^49^, ^50^, ^51^ ^]^ DLP may facilitate the manufacturing of personalized photothermal therapeutic devices by associating functional photopolymers to NIR‐responsive AuNRs in the feedstock formulation. However, the development of DLP 3D printed elastomeric devices for PTT remains challenging due to the lack of suitable biomedical inks.

**Figure 1:**
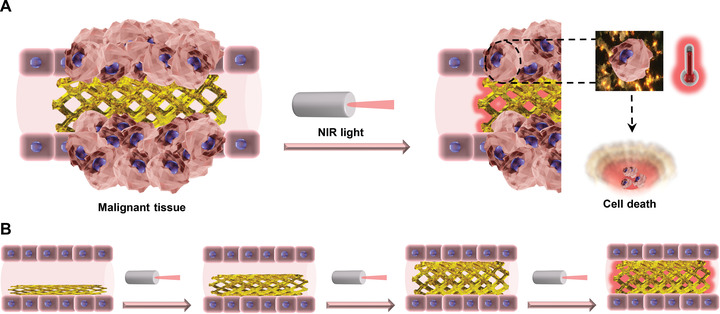
Schematic representation of photothermal therapy and shape‐memory concepts with stent as an exemplary therapeutic device. A) DLP printed bioresorbable stent containing AuNRs inserted in a tubular organ at the place of malignant obstruction. Upon NIR light exposure, AuNRs convert light into thermal energy and induce localized heat‐mediated cell death. B) Stent based on a shape‐memory polymer and AuNRs allowing for facilitated deployment by heat‐mediated shape recovery, while preserving therapeutic effect.

Here, we report the DLP 3D printing of photothermal devices with NIR light‐responsive and elastomeric properties using biomedical inks containing AuNRs and functional polymers (**Figure** **1**A). A highly efficient and reproducible photothermal response was achieved in vitro and ex vivo through the use of optimized resins. Importantly, the developed materials degraded under physiological conditions. Furthermore, the model 3D printed device was designed to feature a shape‐memory function by tuning the photopolymer composition in the DLP biomedical ink. This allowed the fabrication of stents that were easily deployed ex vivo and that expanded upon NIR light irradiation (Figure 1B). This work opens a new route for the development of customized multifunctional photothermal therapeutic devices through the synergistic combination of nanotechnology and 3D printing.

## 2 Results and Discussion

### 2.1 Mechanical and Optical Properties of 3D Printed Composites

**Figure 2:**
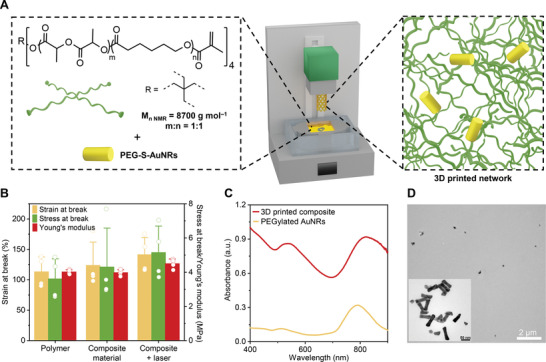
Mechanical and optical properties of 3D printed composite based on poly(DLLA‐*co*‐CL) MA and PEGylated AuNRs. A) Scheme of the strategy for 3D printing of composite materials. B) Average Young's modulus, engineering stress and strain at break of 3D printed composite containing 0.1 wt% of AuNRs (composite material) before and after 3‐min exposure to NIR light. A 3D printed polymer without AuNRs is shown as a control (polymer). Mean + s.d. (*n* = 4). C) UV–vis spectra of PEGylated AuNRs in water and of a 3D printed composite with 0.1 wt% of AuNRs. D) Transmission electron microscopy (TEM) images of the 3D printed composite with 0.1 wt% of AuNRs.

We first synthesized a four‐arm biodegradable photopolymer based on d,l‐lactide (LA) and ɛ‐caprolactone (CL) as the scaffold material for the 3D printed objects. To afford NIR light‐responsiveness to the material, AuNRs functionalized with poly(ethylene glycol) methyl ether thiol (mPEG‐SH) were further incorporated into the biodegradable ink (**Figure** **2**A). The 9k‐copolymer (*M*_n NMR_ 8700 g mol^−1^) was synthesized according to a previously reported procedure, and further end‐caped with methacrylate groups to make it photopolymerizable (Figure S1, Supporting Information).^[^ ^8^, ^52^ ^]^ The statistical distribution of CL and LA, as well as the four‐arm architecture of poly(d,l‐lactide‐*co*‐ɛ‐caprolactone) methacrylate (poly(DLLA‐*co*‐CL) MA) favors low viscosity, which is essential for high‐resolution DLP printing, while relatively high chain length provides ductility to the 3D printed objects.^[^ ^53^, ^54^, ^55^, ^56^ ^]^ AuNRs were synthesized by the seed‐mediated growth method with hydroquinone as the reducing agent.^[^ ^57^ ^]^ The concentrations of silver nitrate and hexadecyl trimethylammonium bromide were varied until the desired optical properties were achieved (Figure S2, Supporting Information). Thereafter, AuNRs were functionalized with mPEG‐SH (*M*_n_ 6000 g mol^−1^) in Tris buffer pH 3^[^ ^58^ ^]^ (Figure S3, Supporting Information) to ensure compatibility with the photopolymer^[^ ^59^, ^60^ ^]^ and the diluent (Figure S4, Supporting Information), thus reducing aggregation in the resin.

To enable high‐resolution printing, the viscosity of the photopolymer was decreased by heating it in a customized DLP printer (75–85 °C) and by adding the reactive diluent *N*‐vinyl‐2‐pyrrolidone (NVP, 15 wt%) to the ink (Figure S5, Supporting Information). NVP also allowed for the dispersion of the PEGylated AuNRs and the dissolution of the photoinitiator (phenylbis(2,4,6‐trimethyl‐benzoyl)phosphine oxide, BAPO, 1.0 wt%), the antioxidant (vitamin E, 0.3 wt%), and the photoabsorber (Sudan I, 0.03 wt%). This resin enabled DLP 3D printing of an amorphous composite (Figure S6, Supporting Information). From the experiments where resins with different amounts of AuNRs were prepared, a concentration of 0.1 wt% was found optimal to provide a rapid photothermal response (Figure S7, Supporting Information) and good mechanical performance (Figure S8, Supporting Information).

Mechanical tests on dog‐bone samples showed that the DLP printed composite displayed excellent elasticity with Young's modulus of 4.0 MPa, elongation at break of 124%, and an ultimate tensile stress of 4.3 MPa (Figure 2B and Figure S8, Supporting Information). As shown in Figure 2B, AuNRs did not affect the mechanical properties of the polymer network, and the impact of NIR light irradiation on the mechanical performance of the composite material was also negligible. Repeated exposure to 808‐nm laser, also resulted in an unchanged mechanical performance (Figure S8, Supporting Information), indicating stability of the polymer matrix toward NIR light. Compared to native AuNRs, the printed composite showed a broader NIR and slightly redshifted absorption with the maximum at ≈815 nm (Figure 2C). This can be explained by the fact that the AuNRs formed some local clusters,^[^ ^59^ ^]^ despite being overall well dispersed in the polymer matrix (Figure 2D). This result also indicates that the PEGylated AuNRs remained stable in the resin even after 3D printing.

### 2.2 Photothermal Performance

**Figure 3:**
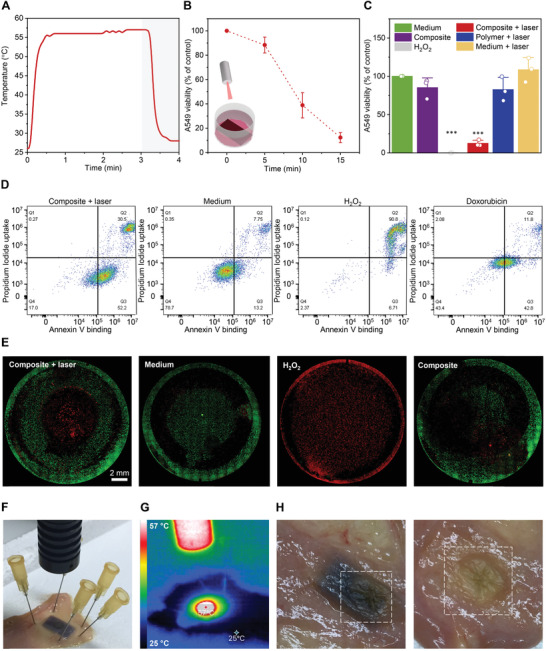
Photothermal performance of 3D printed composites with 0.1 wt% of AuNRs during NIR light irradiation with 808‐nm laser. A) Thermal curve of the 3D printed composite material during 3‐min exposure and 1 min after the discontinuation of the irradiation. B) Decline in cell viability with increased irradiation time of composite‐based 3D printed cuboids (13 × 7 × 0.8 mm). C) Cell viability after 15‐min irradiation of composite‐based 3D printed cuboid (red bar) obtained with MTS assay compared to medium control (green bar). Additional controls including composite‐based 3D printed cuboid without laser irradiation (purple bar), irradiated control material without AuNRs (blue bar), irradiated medium without any material (yellow bar), as well as the positive control (0.1 m hydrogen peroxide, grey bar) are also shown. Mean + s.d. (*n* = 3). Statistical significance was calculated by one‐way analysis of variance (ANOVA) with Tukey's comparison test (^***^ *p* < 0.001). D) Dot plots of flow cytometry analysis with Annexin V and propidium iodide (PI) obtained after 15‐min exposure of composite‐based 3D printed cuboid. Populations were gated based on negative control (medium), positive control for necrosis (0.1 m hydrogen peroxide) and positive control for apoptosis (0.2 µg mL^−1^ doxorubicin hydrochloride) with lower left quadrant corresponding to live (Annexin V and PI negative), upper right quadrant to necrotic (Annexin V and PI positive) and bottom right quadrant to apoptotic cells (Annexin V positive, PI negative). E) Localized photothermal effect demonstrated by live‐dead staining with calcein AM (green, live) and PI (red, dead) in 24‐well plate with 8‐min exposure of composite‐based 3D printed disk (Ø 7.4 mm, thickness 0.8 mm). Additional controls including medium only (live cells), hydrogen peroxide (dead cells), and composite material without laser exposure are also included. F) Ex vivo study with porcine intestine including experimental setup, G) thermal image after 7‐min exposure, and H) tissue images with white squares showing the hardened tissue after 15‐min exposure.

The photothermal efficiency of the DLP printed composite was evaluated based on the temperature increase upon irradiation with an 808‐nm laser at a power density of ≈4.1 W cm^−2^. This laser wavelength was selected to match the SPR peak of AuNRs in the printed material (Figure 2C). The material could be heated up to 55 °C in only 20 s, and remained at the same level within 3 min of laser exposure, indicating fast and stable NIR light‐responsiveness (**Figure** **3**A). Once the light was switched off, the material cooled down to room temperature in 1 min. The same heating profile was observed over three consecutive cycles (Figure 3A and Figure S9, Supporting Information). In addition, the performance remained unchanged during 15 min of continuous irradiation (Figure S9, Supporting Information). Moreover, it was found that a higher maximal temperature can be achieved by increasing the thickness of the material (Figure S9, Supporting Information). This should be taken into consideration during clinical application, as thicker human‐sized stents could shorten the duration of the treatment compared to the thin prototypes reported here. In addition, the observed photothermal response, with 20 s needed for a substantial heating and almost immediate cooling upon removal of the irradiation source should minimize the risk of tissue damage caused by an accidental NIR light exposure. The control 3D printed material devoid of AuNRs showed no increase in temperature over the 3‐min irradiation period (Figure S9, Supporting Information).

Although fast and stable NIR‐light responsiveness is essential, the temperatures generated by the composite in Figure 3A were rather in the range of nonselective irreversible injury than hyperthermia treatment.^[^ ^37^ ^]^ However, the heat transfer from the material to the cells depends on the nature of the surrounding medium. Therefore, we performed a test with a 3D printed cuboid in water at 37 °C. The temperatures ranging from 40 to 45 °C were generated after 5–15 min exposure, respectively (Figure S10, Supporting Information), indicating that in a more physiological environment, the clinically relevant range of temperatures for PTT (41–48 °C) should be achieved.^[^ ^37^, ^61^ ^]^ Subsequently, to evaluate therapeutic potential of the composite material, 3D printed cuboids were placed in close contact with A549 cells and exposed to NIR light. The extent of in vitro cell death increased from 12% to 88% by prolonging the exposure time from 5 to 15 min (Figure 3B). As shown in Figure 3C, the same material without light irradiation did not induce a significant decrease of cell viability when compared to medium control. The same observation held for the material without AuNRs irradiated for 15 min, indicating that the laser exposure itself was not cytotoxic (Figure 3C). After 15 min of irradiation of the AuNRs‐containing composite, apoptosis was identified as the dominant mechanism of cell death, with ≈50% of cell dying by apoptosis and ≈30% by necrosis (Figure 3D). However, with an average of 90% cell confluency and the material covering ≈50% of the surface of the well, the important part of cell death was probably caused by the overheated surrounding medium rather than the spatially controlled thermal effect from the composite itself. Furthermore, overheating of the cells that were placed directly below the part of the material that was directly exposed to NIR light, cannot be excluded. To keep the localized effect, we applied NIR light for 8 min on 3D printed disks (Ø 7.4 mm, thickness 0.8 mm). As shown in Figure 3E, reduced exposure time resulted in a confined cell death (propidium iodide (PI) staining, red) with the size of the dead zone matching the dimensions of the 3D printed disk used in the experiment. A control disk that was in close contact with cells, but without the laser exposure, showed substantially lower cell death (Figure 3E). To further improve the selectivity, one could conceive a printed device having a distinct composite‐based NIR light‐responsive section that would be in contact with malignant tissue, while the non‐heating polymer‐only‐based parts would spare the healthy cells.

The photothermal performance of the 3D printed composite was further investigated by performing an ex vivo heating test with a porcine intestine (Figure 3F). The 3D printed composite material was placed underneath the tissue and exposed to the 808‐nm laser, causing an increase of temperature from 25 to 57 °C after 7 min (Figure 3G). After 15 min of exposure, the tissue appeared to be deeply burned and hardened (Figure 3H). In a clinical setting, the NIR light exposure time could be shortened and AuNRs concentration in the device decreased to achieve temperatures in the PTT range. However, only preclinical in vivo studies would show how heat is dissipated in the anatomical region where the stent is inserted and which temperatures can be reached. In addition, concentration of AuNRs as low as 0.1 wt% was enough to provide radiopacity to the composite material, making the stent visible under X‐ray in an ex vivo study with a rabbit's cadaver (Figure S11, Supporting Information).

### 2.3 Cytocompatibility and Degradation

**Figure 4:**
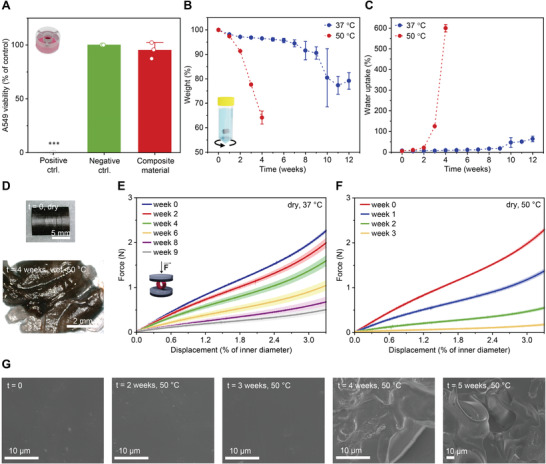
Cytocompatibility and degradation of 3D printed composites with 0.1 wt% of AuNRs. A) Cell viability obtained with MTS assay. Polymer composite (red bar) was compared to medium control (green bar). Positive control (0.1 m hydrogen peroxide) is shown as a gray bar. Mean + s.d. (*n* = 3). Statistical significance was assessed by one‐way ANOVA with Tukey's comparison test (^***^ *p* < 0.001). B,C) Changes in dry weight and water uptake of 3D printed stents (H 10.0 mm, Ø 8.0 mm, thickness 1.0 mm) during incubation in MOPS buffer pH 7.4 at 37 and 50 °C. Mean ± s.d. (*n* = 4). D) A representative stent in a dry state before the degradation study and its disintegration in a wet state after four weeks at 50 °C. E,F) Average compression curves of dried stents during incubation at 37 and 50 °C. Mean ± s.d. (*n* = 4). G) SEM images of cuboids (4 × 3 × 1 mm) in a dry state over five weeks of degradation at 50 °C.

In addition to good mechanical properties and NIR‐responsiveness, the composite devices for photothermal therapy should also have a good safety profile. To assess the cytocompatibility, composite‐based disks were thoroughly cleaned and placed on top of Transwell supports. Cell viability was then determined after keeping samples fixed above A549 cells for 48 h.^[^ ^8^ ^]^ No significant change in cell viability was observed compared to the control medium, indicating cytocompatibility of the composite material (**Figure** **4**A).

Biodegradability is another main feature of the designed DLP‐printed composite. To assess this property, we investigated the degradation profile of the material using 3D printed stent‐like objects incubated in 3‐(*N‐*morpholino)propanesulfonic acid (MOPS) buffer pH 7.4 at 37 or 50 °C. At physiological temperature, the printed material showed autocatalytic bulk degradation,^[^ ^62^ ^]^ with a slow decline over seven weeks, followed by a more rapid mass loss (Figure 4B). This is typical for polyester‐based materials, where the products of hydrolysis stay entrapped in the polymer network and catalyze the degradation.^[^ ^52^, ^62^, ^63^ ^]^ As shown in Figure 4C, the water uptake remained low (7–18 wt%) during the first nine weeks, reflecting the initial slow diffusion. In the accelerated degradation study at 50 °C, the material rapidly lost as much as 36 wt% of its initial mass after four weeks. The water uptake reached 600 wt% at that time, transforming the stents into soft hydrogels (Figure 4B–D) that should, in principle, not induce any mechanical damage to the tissue.^[^ ^64^ ^]^

Despite the favorable degradation kinetics, bioresorbable medical devices still need to provide mechanical support over the duration of the treatment. We evaluated the mechanical performance of the device during degradation by measuring the load‐bearing capacity of model stents under uniaxial compression. The results showed that the dried 3D printed stents gradually lost their uniaxial compression resistance due to the hydrolysis of the ester bonds. As shown in Figure 4E, ≈50% loss of resistance was observed after six weeks of incubation at 37 °C, while the mass and shape remained unchanged (Figure 4B). As stents are usually in contact with wet environment, the uniaxial compression curves were also recorded in a wet state. The experiments revealed no difference at the same time point between wet and dry stents over the testing period (Figure S12, Supporting Information). In the accelerated degradation study, the uniaxial compression resistance decreased much faster than that at physiological temperature (Figure 4F). This results from the higher rate of hydrolysis caused by elevated temperature and water uptake (Figure 4C). As shown on scanning electron microscopy (SEM) images in Figure 4G, the surface morphology of the 3D printed material remained largely intact until it transformed into a hydrogel after 4 weeks of incubation at 50 °C, confirming that the shape of the 3D printed object can be preserved for a certain time. This can be explained by the fact that in autocatalytic bulk degradation surface erodes slowly because the released degradation products are buffered by the surrounding medium.^[^ ^65^, ^66^ ^]^ Together with the swelling, this is important to prevent devices such as nonvascular stents from migrating away from their insertion site. Furthermore, elemental gold could be detected by energy dispersive X‐ray (EDX) analysis over the whole degradation period (Figure S13, Supporting Information), while inductively coupled plasma mass spectrometry (ICP‐MS) analysis revealed that the composite stents release less than 0.2% of theoretical gold amount during four weeks of accelerated degradation (Figure S14, Supporting Information). These results confirm the stability of the incorporated AuNRs, which could be of practical relevance if therapeutic protocols would include several cycles of NIR light administration over several weeks. Although mechanical properties of the stents remained stable over several cycles of 808‐nm laser exposure (Figure S8, Supporting Information), contribution of NIR light to the degradation process should be further evaluated. The degradation would depend on the laser power, exposure time, and treatment frequency.

### 2.4 Shape‐Memory Properties

The proposed strategy for designing DLP biomedical inks allows for the introduction of additional functionalities to the 3D printed materials by adjusting the composition of the copolymer and the ligand for AuNRs. To demonstrate this capability, we printed stents of various sizes with a modified polymer composition in order to confer shape‐memory properties.

**Figure 5:**
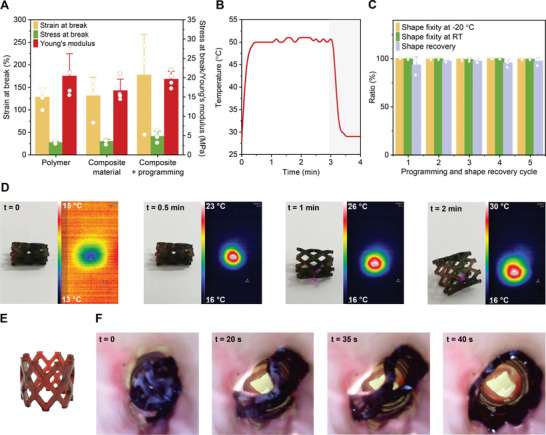
Mechanical, photothermal, and shape‐memory properties of 15k‐based 3D printed composites with 0.1 wt% of AuNRs. A) Average Young's modulus, engineering stress and strain at break of 3D printed composite material before and after programming step, as well as of polymeric control without AuNRs. Mean + s.d. (*n* = 4). B) Thermal curve during 3‐min exposure and 1 min after the discontinuation of the irradiation. C) Shape fixity (*R*_f_) at −20 °C and at room temperature, and shape recovery (*R*_r_) ratios of shape‐memory cuboids (12.5 × 2.4 × 0.8 mm) over five loading cycles. The deformed shape was preserved at −20 °C for 10 min and *R*_f_ was determined 2 min after the removal of external force at −20 °C and after additional 2 min at room temperature. *R*_r_ was calculated after 1 min of exposure to 808‐nm laser. Mean + s.d. (*n* = 3). D) Optical and thermal images of a meshed stent (H 11 mm, Ø 10.8 mm, and thickness 1.1 mm) DLP printed with shape‐memory polymer‐AuNRs ink after the programming step (heating–folding–freezing) upon exposure to 808‐nm laser at different time points. The experiment was performed at ≈16 °C. E) Composite‐based 3D printed stent in the human size (H 12 mm, Ø 16 mm, and thickness 1.6 mm). F) Stent after the programming step and its shape recovery ex vivo in the porcine intestinal segment upon NIR light irradiation.

To create shape‐memory printed devices, we synthesized 15k poly(DLLA‐*co*‐CL) MA (*M*_n NMR_ 15 200 g mol^−1^) with a molar ratio of CL:LA of 9:1 (Figure S1, Supporting Information).^[^ ^8^, ^52^ ^]^ The higher molecular weight and the molar fraction of CL compared to the initial 9k‐photopolymer was aimed at obtaining a semicrystalline material with a melting point close to the body temperature (*T_m_* = 39.2 °C, Figure S15, Supporting Information). To prepare stable AuNRs compatible with the shape‐memory copolymer, a new thiolated polymer ligand, poly(DLLA‐*co*‐CL)‐SH, was also synthesized (Figure S16, Supporting Information) and used to coat the AuNRs. The ligand had the same molar ratio of CL to LA as the 15k‐copolymer and *M*_n NMR_ similar to the initial mPEG‐SH coating agent. The ligand exchange reaction on AuNRs was performed in tetrahydrofuran (THF), as described previously.^[^ ^67^ ^]^ The SPR longitudinal peak of AuNRs remained in the NIR light region after the functionalization (Figure S17, Supporting Information). The 15k‐shape‐memory polymer was then formulated in a same way as the 9k‐photopolymer, by using 15 wt% of NVP in the resin to reduce its viscosity to the printable range at 75–85 °C (Figure S18, Supporting Information). The melting point of the 15k‐photopolymer decreased by ≈6 °C after 3D printing (Figure S15, Supporting Information) due to the presence of NVP in the crosslinked network.^[^ ^68^ ^]^ The 3D printed 15k‐composite was less elastic than the 9k‐composite (Figure 2B), with Young's modulus of ≈16.6 MPa, and similar elongation at break and ultimate tensile stress of ≈131% and 3.5 MPa, respectively (**Figure** **5**A and Figure S19, Supporting Information). The 9k‐based materials demonstrated elasticity similar to silicone which is widely used for prototyping devices for biomedicine and has Young's modulus of ≈1 MPa,^[^ ^69^ ^]^ while 15k one is similar to a commercial silicone airway stent with Young's modulus of ≈13 MPa.^[^ ^8^ ^]^ Our materials were also compared in cyclic creep experiments with 50% elongation. Composite based on 9k polymer showed permanent deformation of 0.5%–0.9%, which is very close to an ideal elastomer (Figure S20, Supporting Information). On the contrary, 15k‐based composite was permanently deformed to 10.7%–14.0%, indicating plastic deformation (Figure S20, Supporting Information). Although materials for nonvascular stents would be exposed to certain stresses over long period of time, they should not experience such severe load‐bearing conditions and related deformations in a clinical setting. In addition, shape‐memory materials will be substantially more elastic at physiological temperature, which is reflected by the modulus drop in dynamic mechanical analysis (DMA) (Figure S21, Supporting Information). As expected, the incorporation of AuNRs did not change the mechanical properties of the 3D printed polymer network, nor did the programing step (Figure 5A). Uniaxial compression test with stent‐like structures after 3D printing and after three cycles of deformation with NIR light‐induced shape recovery was also performed (Figure S19, Supporting Information). Normal stiffness decreased by 25% (Figure S19, Supporting Information), probably due to the melting of certain domains in polymer network that did not have enough time to recrystallize over several consecutive loading cycles. This indicates that the time between the shape‐memory cycles should be prolonged, to allow materials to fully recover their mechanical performance. The optical properties of AuNRs were preserved after 3D printing (Figure S17, Supporting Information), which resulted in heating of the materials up to 50 °C after 1 min of exposure to 808‐nm laser (Figure 5B and Figure S22, Supporting Information).

Shape‐memory properties were evaluated based on shape fixity (*R*_f_) and shape recovery (*R*_r_) ratios of printed cuboids before and after five cycles of deformation at 80 °C, preservation of the deformed shape at −20 °C, and recovery to the initial form during 1‐min exposure to 808‐nm laser. Based on the change in angles of the cuboids (Figure S23, Supporting Information), average *R*_f_ after 2 min at −20 °C (*R*_f −20 °C_) and room temperature (*R*_f RT_), as well as *R*_r_ were calculated to be in a range of 97.6%–100%, 97.0%–100%, and 83.1%–100%, respectively, over five consecutive cycles (Figure 5C). The material preserved its shape‐memory properties over five loading cycles, implying that the mobility of polymer chains within the crosslinked structure can be altered and recovered in a similar manner when repeatedly applying the same temperature program. High shape fixity ratio at room temperature (Figure 5C), as well as the unchanged programmed shape of the control sample over 2 days (Figure S23, Supporting Information) indicated that the 15k‐based devices could be applied at room temperature without the risk of premature shape recovery. As NIR‐light is inducing heat generation, we also performed DMA to more precisely characterize the shape‐memory behaviour. Shape fixity ratio at 4 °C and shape recovery ratio at 37 °C were 97.1% and 92.5%, respectively (Figure S24, Supporting Information), which is in accordance with the results obtained by angle measurements (Figure 5C).

Using the shape‐memory polymer‐AuNRs ink, two meshed stents were fabricated by DLP and their NIR light‐triggered shape recovery was assessed. To fix a temporary shape, the stent was first folded above its transition temperature and subsequently cooled quickly to −20 °C. A test with a small non‐vascular stent (H 11 mm, Ø 10.8 mm, and thickness 1.1 mm) was then performed in a temperature‐controlled cold room to minimize the environmental impact on shape transformation (Figure 5D). Upon exposure to a laser light of 808 nm, the composite‐based stent started opening after 1 min when the temperature reached 26 °C, and fully recovered after 2 min when heated up to 30 °C (Figure 5D). As the material has a *T_m_* of ≈33.5 °C (Figure S15, Supporting Information), it started to expand slowly at 26 °C. However, the shape recovery was much faster when the temperature approached the transition point, which is required for a quick expansion after stent insertion. The control stent without AuNRs did not open even after 5 min of irradiation (Figure S25, Supporting Information), confirming that the photothermal performance of the material can be attributed to the AuNRs. However, as the spot size of the laser beam used in these experiments was small, the light needed to be directed to different parts of the stents in order to entirely recover the initial shape, which prolonged the duration of the shape recovery process. Therefore, the stent should first be positioned with the help of a balloon to keep it at the targeted place. Then, a laser fiber optic light‐guide should go through the lumen of the balloon^[^ ^70^ ^]^ and the applied light should trigger heat generation that will first uniformly and rapidly open the stent and then affect the cancerous cells. This way, the treatment time would be shortened. Afterward, the ballon would be deflated and removed.

To investigate the shape recovery in a setup closer to the clinical application, a meshed stent in a human size (H 12 mm, Ø 16 mm, and thickness 1.6 mm) was 3D printed (Figure 5E) and inserted in a porcine intestinal segment. The stent was deployed in a folded form and NIR light of 808 nm was shined from the outer side of the intestine (Figure S26, Supporting Information). Upon exposure to NIR light, the stent heated up rapidly, recovering to its initial 3D printed shape and opening the intestine in only 40 s (Figure 5F and Video S1, Supporting Information). This demonstrates the potential of the shape‐memory function for on‐demand deployment and rapid NIR light‐triggered expansion of biodegradable stents.

Future work with NIR light‐responsive shape‐memory devices should also address their mechanical performance in vivo, due to the change in flexibility when the temperature exceeds their transition point.^[^ ^71^ ^]^ For example, the reported material with *T_m_* of 33.5 °C remained elastic, but showed ≈3 times lower normal stiffness at 37 °C than at room temperature (Figure S27, Supporting Information). If stronger mechanical support is required for a specific medical application, building blocks and architecture of shape‐memory polymers^[^ ^71^ ^]^ could be optimized to shift the transition point of the DLP‐printed network to higher temperatures. Any composite stent with functionalized AuNRs compatible with the polymer matrix and transition point below 57 °C (Figures 3A and 5B) could be suitable for NIR light‐triggered expansion. Furthermore, the forces exhibited by the device will heavily depend on its thickness, diameter,^[^ ^8^ ^]^ and design (Figure S27, Supporting Information), which should also be considered when manufacturing a device for an individual patient and a specific medical indication.

## 3 Conclusion

We developed biomedical inks based on AuNRs‐polymer composites using poly(DLLA‐*co*‐CL) MA and AuNRs functionalized with thiolated polymers that allow for high‐resolution 3D printing of NIR light‐responsive elastomers suitable for photothermal therapy. Photothermal performance and related cell death were demonstrated through the rapid and stable heating of the printed material upon NIR light irradiation. The photo‐responsive elastomer could also be tuned to achieve shape‐memory properties and thus facilitate the insertion of printed stents by NIR light‐triggered shape recovery. Furthermore, 3D printed stent‐prototypes exhibited in vitro degradability under physiological conditions, and eventually disintegrated to harmless hydrogels. This is particularly important for oncology patients, as these stents would disappear after they fulfill their supportive and therapeutic roles, eliminating the need for reinterventions to remove them. Despite their promising potential, the proposed composites should still be optimized for a specific application, which could require materials with a higher stiffness. This could be achieved by, for instance, increasing crosslinking density via the incorporation of crosslinkable oligomers.^[^ ^8^ ^]^ Although the focus of this study was on photothermal stents, the proposed strategy could be used to develop a wide range of high‐performance personalized medical devices that combine NIR‐responsiveness, bioresorbability, and shape‐memory features.

## 4 Experimental Section

### Materials

Pentaerythritol, methacryloyl chloride, triethylamine, THF (extra dry), acetone (extra dry), and hydrogen peroxide (35 wt% in water) were purchased from Acros Organics. CL, hydroquinone, sodium dodecyl sulphate (SDS), and bis(2‐hydroxyethyl) disulfide were obtained from Tokyo Chemical Industry. 3,6‐Dimethyl‐1,4‐dioxane‐2,5‐dione (LA) was purchased from Huizhou Foryou Medical Devices Co., Ltd. or Acros Organics. Tin(II)‐2‐ethylhexanoate (Sn(Oct)_2_), (+)‐*α*‐tocopherol (vitamin E), NVP, BAPO, 1‐(phenyldiazenyl)naphthalen‐2‐ol (Sudan I), hexadecyltrimethylammonium bromide (CTAB), sodium borohydride (NaBH_4_), mPEG‐SH (average *M*_n_ 6000 g mol^−1^), tris(hydroxymethyl)aminomethane (Tris), MOPS, sodium hydroxide (NaOH), chloroform‐d (CDCl_3_), d,l‐dithiothreitol (DTT), sodium methoxide solution (25 wt% in methanol), and lithium bromide (LiBr) were purchased from Sigma Aldrich. Hydrogen tetrachloroaurate(III) trihydrate (HAuCl_4_) was obtained from abcr. Silver nitrate (AgNO_3_) was purchased from Strem Chemicals, Inc. Hexane and dimethylformamide (DMF) were purchased from Fisher Scientific. Dimethyl sulfoxide‐d6 (DMSO‐d6) was obtained from Apollo Scientific. Methanol and 2‐propanol were provided by VWR Chemicals. Calcein‐AM was purchased from Cayman Chemical. PI was obtained from Chemodex. Dead Cell Apoptosis Kit with Annexin V FITC and PI was provided by Thermo Fisher Scientific. All chemicals were used as received.

### Polymer Synthesis

Poly(DLLA‐*co*‐CL)s were synthesized by ring‐opening polymerization of LA and CL with pentaerythritol as the initiator and Sn(Oct)_2_ as a catalyst at 140 °C for 48 h (Figure S1, Supporting Information).^[^ ^8^, ^52^ ^]^ After purification in hexane and drying, the conversions of LA and CL as determined by ^1^H nuclear magnetic resonance (^1^H NMR) spectroscopy were ≈90% and 99% for 9k‐polymer and ≈91%–94% and 98%–99% for 15k‐polymer, respectively (Figure S28, Supporting Information). The polymers showed low polydispersity by size exclusion chromatography (SEC) (Table S1, Supporting Information). Afterward, poly(DLLA‐*co*‐CL)s were dissolved in extra dry THF and functionalized with methacryloyl chloride in the presence of triethylamine at room temperature for 24 h (Figure S1, Supporting Information).^[^ ^8^, ^52^ ^]^ After purification in methanol and subsequent drying, the conversion of hydroxyl end groups of the polymer chains was ≈78%–85% and 64%–74% for 9k‐polymer and 15k‐polymer, respectively, as determined by ^1^H NMR spectroscopy (Figure S29, Supporting Information).

### Polymeric Ligand Synthesis

Poly(DLLA‐*co*‐CL) disulfide was synthesized by ring‐opening polymerization of LA and CL with bis(2‐hydroxyethyl) disulfide as the initiator and Sn(Oct)_2_ as a catalyst at 100 °C for 24 h (Figure S16, Supporting Information).^[^ ^8^, ^72^ ^]^ After purification in hexane and drying, the conversions of LA and CL as determined by ^1^H NMR spectroscopy were ≈96% and 95%, respectively (Figure S30, Supporting Information). Afterward, poly(DLLA‐*co*‐CL) disulfide was dissolved in THF together with DTT and sodium methoxide (25 wt% in methanol) was added as a catalyst at room temperature and the reaction was stirred for 72 h (Figure S16, Supporting Information).^[^ ^73^ ^]^ The solution was concentrated and precipitated in methanol. After purification, poly(DLLA‐*co*‐CL)‐SH was obtained (Figure S30, Supporting Information). The synthesized polymers showed low polydispersity which was confirmed by SEC (Table S2, Supporting Information).

### Polymer Characterization

^1^H NMR spectra were recorded on Bruker AV400 spectrometer at 400 Hz using DMSO‐d6 or CDCl_3_ as solvents. SEC was performed on a Viscotek TDAmax system with two Viscotek columns (D3000, poly(styrene‐*co*‐divinylbenzene)) and differential refractive index detector (TDA 302, Viskotek). All samples were dissolved in DMF, filtered through 0.2 µm syringe filters (polytetrafluoroethylene, PTFE) and eluted by DMF with LiBr (0.1 wt%) of 0.5 mL min^−1^ flow rate. The properties were calculated relative to a poly(methyl methacrylate) (PMMA) standard curve (PSS polymer Mainz, 2500–89 300 g mol^−1^). Differential scanning calorimetry (DSC) was carried out on a TA Q200 DSC (TA Instruments–Waters LLC). The samples (≈10 mg) were placed on Tzero hermetic pans (TA Instruments–Waters LLC) and exposed to heat–cool–heat cycles from −80 to 200 °C under nitrogen flow (50 mL min^−1^) using heating and cooling rates of 10 °C min^−1^. Data were analyzed using TA Instruments Universal Analysis 2000 software (5.5.3). Viscosity measurements were completed on HAAKE RheoStress 600 rotational rheometer (Thermo Electron Corporation) with cone and plate geometry (35 mm/2°). Viscosity was determined at 60–90 °C with a shear rate of 100 s^−1^ and a heating rate of 0.05 °C s^−1^ with ThermoGap function enabled. Data were analyzed by RheoWin Data Manager (Thermo Electron Corporation). DMA was conducted on DMA861^e^ (Mettler Toledo) in nitrogen atmosphere by applying a constant frequency of 3 Hz and an oscillation strain of 0.5%. The instrument was equipped with sheering clamps and disk‐like samples (Ø 5.2 mm and thickness 2 mm) were used. The temperature was ramped at 2 °C min^−1^ from −50 to 100 °C and the storage (*G*′) and loss (*G*″) moduli were monitored.

### Gold Nanorods Synthesis

Water solution of HAuCl_4_ (5 mL, 0.5 × 10^−3^ m) was added to 5 mL of the CTAB (364 mg) solution. Afterward, NaBH_4_ solution (600 µL, 10 × 10^−3^ m) was added to the mixture and stirred for ≈20 min until the gold seeds were formed. Then, AgNO_3_ water solution (4 mL, 4 × 10^−3^ m) and HAuCl_4_ solution (1 × 10^−3^ m, 100 mL) were added subsequently to 100 mL of CTAB (3.644 g) and hydroquinone (440 mg) solution. Shortly after, the gold seeds (240 µL) were added and the mixture was stirred at 27 °C for 1.5 h until the formation of the AuNRs. The suspension was centrifuged (4000 × *g*, 27 °C, 50 min), and the pellet was resuspended in 4 mL of water and stored at room temperature. All the solutions were prepared in nanopure water.^[^ ^57^ ^]^ For PEGylated AuNRs, AuNRs (≈20 mg) were centrifuged and the pellet was functionalized with mPEG‐SH (average *M*_n_ 6000 g mol^−1^, 165 mg) in Tris buffer pH 3 (4 mL, 50 × 10^−3^ m) on an orbital shaker at room temperature for 1 h. To remove the excess of CTAB, the suspension was centrifuged again and the pellet was dissolved in 1240 µL of NVP.^[^ ^58^ ^]^ For AuNRs functionalized with poly(DLLA‐*co*‐CL)‐SH, AuNRs (≈10 mg) were centrifuged, the pellet was added to the THF solution (50 mL) of poly(DLLA‐*co*‐CL)‐SH (606 mg) and the mixture was stirred at room temperature for 72 h. After centrifugation, the pellet was dissolved in 620 µL of NVP.^[^ ^67^ ^]^

### Gold Nanorods Characterization

UV–visible spectroscopy was carried out on Cary 60 Bio UV‐Vis spectrophotometer with CaryWinUV software (Agilent). Spectra were obtained from 350 to 900 nm with a distilled water solution of gold seeds, AuNRs, and functionalized AuNRs, as well as with the pieces of the 3D printed composite materials. TEM images of AuNRs in distilled water solutions (≈0.01 mg mL^−1^) with different CTAB concentrations were captured on scanning/transmission electron microscope FEI Talos F200X (Thermo Fisher Scientific) with 100 kV acceleration voltage. TEM images of cross sections of 3D printed object were obtained on transmission electron microscope JEOL JEM‐1400 (JEOL) after cryosectioning on Leica FC6 cryo‐ultramycrotome (Leica Microsystems).

### DLP 3D Printing

Resins were prepared by adding vitamin E (antioxidant, 0.3 wt%) and a solution of BAPO (photoinitiator, 1.0 wt%) with Sudan I (photoabsorber, 0.03 wt%) in NVP (reactive diluent, 7.5 wt%) to the photopolymer. The resins were sonicated at 60 °C until homogeneity was achieved. Then, AuNRs (0.07, 0.13, or 0.19 wt%) in NVP (7.5 wt%) were added and vigorously mixed without further heating. Reported wt% of AuNRs in the printed materials refers to wt% of gold, as the weight of functionalized AuNRs could not be determined. For control materials, resins with 15 wt% NVP without AuNRs were used. A DLP 3D printer (Asiga PICO2) with the LED light source of 405 nm and in‐house modifications enabling heating of the resin tray and the printing head was used to manufacture all the objects. The printing was performed at 85 °C, with exposure time of 3.3 s and burn‐in exposure of 15 s. After the printing, the printed objects were washed in acetone and 2‐propanol, dried, and then cured in an Asiga Pico Flash UV chamber for 15 min.

### Mechanical Characterization of 3D Printed Materials

Tensile tests were performed on a dog‐bone specimen at a rate of 20 mm min^−1^ with a gauge length of 13 mm using an AGS‐X (Shimadzu) universal testing machine with a 100‐N load cell. Young's modulus was calculated as the slope of the initial part (first 10 points) of the engineering stress–strain curve. Compression tests were performed on stent‐like 9k‐based 3D printed specimens (H 10 or 5 mm, Ø 8 mm, thickness 1 mm) at a rate of 12 mm min^−1^ using TA.XTplus texture analyzer (Stable Micro Systems) with 100‐N load cell. Compression tests with stent‐like 15k‐based 3D printed specimens (H 7.2 mm, Ø 4.6 mm, thickness 0.85 mm) were performed at a rate of 0.01 mm s^−1^ using AresG2 DMA instrument (TA Instruments). For compression at 37 °C, a nitrogen‐purged oven was used and the samples were incubated for 2 min prior to test. Every sample was compressed three times. Cyclic creep experiments were performed on 9k‐ and 15k‐based dog‐bone specimens at a rate of 5 mm min^−1^ with a gauge length of ca. 13 mm using an AGS‐X (Shimadzu) universal testing machine with a 100‐N load cell. The samples were stretched to 50% strain over 20 cycles. The permanent deformation was determined after 2 h of recovery as the difference in strain between 1st and 21st cycle at a force of 0.05 N (the lowest limit of the instrument).^[^ ^74^ ^]^ The test was performed in duplicates for each composition.

### In Vitro Degradation Study

The 3D printed stent‐like objects (H 10.0 mm, Ø 8.0 mm, thickness 1.0 mm) were divided in two groups of four specimens and every object was immersed separately in 50 mL MOPS buffer (pH 7.4, 0.1 m) at 37 or 50 °C in closed containers. MOPS buffer was selected over commonly used phosphate saline buffer (PBS) to avoid presence of high concentration of phosphorus, which negatively impacts ICP‐MS analysis of elemental gold. This analysis was needed to estimate release of AuNRs during accelerated degradation. The medium was replaced weekly and at each time point, the specimens were taken out, rinsed with deionized water, wiped with paper tissue and dried under vacuum at 50 °C to constant weight. The water uptake (wt%) was calculated using Equation (1)

(1) $$\mathrm{water}\;\mathrm{uptake}=\frac{wt_{\mathrm{wet}}-wt_{\mathrm{dry}}}{wt_{\mathrm{dry}}}\times \;100$$

where *wt*_wet_ is the mass of a stent in a wet state after the wiping and *wt*_dry_ is the mass of a stent in a dry state. Uniaxial compression test was performed with objects in wet and dry states incubated at 37 °C, and with dry objects tested at 50 °C. Objects in accelerated study were visualized using a Keyence scanning laser microscope (Keyence Corporation).

The change in surface morphology of the polymer matrix incubated at 50 °C (3D printed cuboids, 4 × 3 × 1 mm) was observed by a field emission scanning electron microscope (JSM 7100F, JEOL GmbH) in secondary electron mode. The samples were rinsed with deionized water, lyophilized, and then mounted on metallic stubs using carbon tape and coated with 5 nm carbon (CCU‐010, Safematic GmbH). Cross‐section images of the printed object were obtained at an acceleration voltage of 2 kV. The presence and distribution of gold in the printed object was assessed by EDX (Ametek‐EDAX using a Si(Li) 30 mm^2^ detector) at an acceleration voltage of 12 kV.

The release of gold from the polymer matrix in the accelerated degradation study was quantified by ICP‐MS (QQQ 8900, Agilent) in the analytical lab at EPFL (Switzerland). Each sample was diluted in 1% HNO_3_ (1:1, v/v) prior to ICP‐MS measurement. The calibration curve was created in a range from 0.5 to 20 µg mL^−1^ of Au in 1% HNO_3_. Relative release was calculated based on the theoretical values of incorporated AuNRs.

### Cell Culture

Human lung epithelial cells (A549, ATCC) were cultured at 37 °C in a humidified atmosphere with 5% CO_2_. The cells were used up to passage number 22 and were tested for mycoplasma contamination (MycoAlertTM Mycoplasma Detection Kit, Lonza) at first and last passage number. They were cultured in complete medium DMEM (high glucose, GlutaMax, pyruvate) with 10% fetal bovine serum and 1% penicillin‐streptomycin (Thermo Fisher Scientific).

### In Vitro Cytocompatibility and Photothermal Performance Tests

The tests were performed in 24‐well plates with seeding density of 50 000 cells per well in three independent experiments with three replicates. For positive and negative control, cells were incubated in complete medium with 0.1 m hydrogen‐peroxide and complete medium only, respectively. Cell viability was calculated based on the results of the 3‐(4,5‐dimethylthiazol‐2‐yl)‐5‐(3‐carboxymethoxyphenyl)‐2‐(4‐sulfophenyl)‐2H‐tetrazolium (MTS) assay (CellTiter 96 Aqueous One Solution Cell Proliferation Assay, G3580; Promega) as a percentage of the negative control. Prior the incubation with the cells, all the objects were cleaned as described in our previous work.^[^ ^8^ ^]^ In cytocompatibility study, 3D printed disks (H 0.8 mm, Ø 5.5 mm) were placed on Transwell permeable supports (ThinCert Cell Culture Inserts 24‐well, sterile, translucent, pore size 8 µm; Greiner Bio‐One) and inserted above the seeded cells. On top of the inserts, 100 µL of medium was added and the plate was incubated for 48 ± 1 h. For photothermal performance study, the first test was perfomed with a cuboid (13 × 7 × 0.8 mm) placed in 600 µL of water. The material was then exposed to a 808‐nm laser (Fuzhe) over 15 min from 3.5 cm distance at 37 °C. A thermocouple (K type, National Instruments) was immersed in water and the temperature was followed over time. Afterward, cuboids of composite or control materials were in close indirect contact with the cells (seeded as described above) for 5, 10, or 15 min and then removed before MTS assay. The selected cuboids or wells were exposed to a 808‐nm laser (Fuzhe) from 3.5 cm distance at 37 °C, without temperature measurement. Laser power density for the given distance was calculated by dividing the laser power with the surface of the laser beam's spot and it was found to be ≈4.1 W cm^−2^. The laser power was measured with S405C thermal power sensor (Thorlabs), while the surface of the laser spot was obtained from an image by ImageJ software (National Institutes of Health).

### Mechanism of Cell Death

A549 cells were seeded in 24‐well plates with seeding density of 50 000 cells per well. After 24 h of incubation at 37 °C in a humidified atmosphere with 5% CO_2_, clean cuboids (13 × 7 × 0.8 mm)^[^ ^8^ ^]^ were added to the selected wells and exposed to a 808‐nm laser (Fuzhe) for 15 min from 3.5 cm distance at 37 °C. Afterward, medium was removed, cells were washed with PBS, and then detached by trypsinization, followed by the addition of the removed medium to stop the activity of trypsin. The collected medium with PBS was centrifuged (300 × *g*, 4 °C, 10 min) and the supernatant was discarded. The pellet was resuspended with cold PBS and centrifuged again. The sedimented cells were then resuspended in 100 µL of binding buffer and 5 µL of annexin with 1 µL of PI was added to the cell suspension and then incubated in dark for 15 min. Afterward, 200 µL of binding buffer was added and the cell suspension was transferred to the wells. Data for 10 000 cells were collected on a CytoFLEX Flow Cytometer (Beckman Coulter Inc.) and analyzed using the FlowJo software (Tree Star Inc.).

### Live/Dead Staining

A549 cells were seeded in 24‐well plates with seeding density of 50 000 cells per well. After 24 h of incubation at 37 °C in a humidified atmosphere with 5% CO_2_, medium was removed and cells were washed three times with PBS. Afterward, the cells were incubated for 30 min with a solution of 2 × 10^−6^ m of calcein‐AM and 3 × 10^−6^ m of PI in DMEM without phenol red (360 µL per well). Cleaned 3D printed disks (Ø 7.4 mm, thickness 0.8 mm)^[^ ^8^ ^]^ were placed in the close indirect contact with cells and the selected wells were exposed to a 808‐nm laser (Fuzhe) for 8 min from 3.5 cm distance at 37 °C. Cells in the complete medium and in 0.1 m hydrogen peroxide were used as negative and positive control, respectively. Afterward, the fluorescence images were recorded by DMI6000B wide field fluorescence microscope (Leica microsystems) and processed by LAS X software (Leica microsystems).

### Shape‐Memory Properties

Three cuboids (12.5 × 2.4 × 0.8 mm) were evaluated for their shape‐memory properties over five consecutive shape‐memory cycles. Each cycle consisted of heating a sample to 80 °C for 2 min, deforming it and placing it in a 200 µL pipette tip. The sample was then kept at −20 °C for 10 min and the maximal angle (*θ*_max_) was captured by a phone camera. Afterward, the external force was removed and the sample was kept at −20 °C for 2 min, which was recorded as a fixed angle (*θ*_fixed −20 °C_). The sample was left at room temperature for additional 2 min and the fixed angle (*θ*_fixed RT_) was captured. Then, the sample was exposed to NIR light (808‐nm laser) for 1 min and the unrecovered angle (*θ*_unrecovered_) was recorded.^[^ ^17^ ^]^ In case a sample flipped to the other side during heating, it was manually brought back to its initial position. For a control sample, instead of repeated NIR light exposure, sample was left for 2 days at room temperature. Shape fixity ratio at −20 °C (*R*_f −20 °C_) and at room temperature (*R*_f RT_), as well as shape recovery ratio (*R*_r_) were calculated using Equations (2)–(4), respectively

(2) $$R_{f\;-20{\;}^{\circ }C}\;=\;\frac{\theta_{\mathrm{fixed}\;-20{\;}^{\circ }C}}{\theta_{\max}}\;\times \;100$$

(3) $$R_{f\;\mathrm{RT}}=\;\frac{\theta_{\mathrm{fixed}\;\mathrm{RT}}}{\theta_{\max}}\;\times \;100$$

(4) $$R_{r}=\;\frac{\theta_{\max}-\theta_{\mathrm{unrecovered}}}{\theta_{\max}}\;\times \;100$$

DMA was performed on ARES‐G2 (TA Instruments) in nitrogen atmosphere with dog bone‐shaped specimens. The instrument was equipped with an environmental test chamber and a torsion fixture. The sample was first heated to 65 °C with a heating rate of 2.5 °C min^−1^ and then stretched with a constant force of 0.3 N for 3 min. Afterward, the temperature was decreased to 4 °C at the cooling rate of 2.5 °C min^−1^ and then kept at 4 °C for 5 min (ɛ_max_), while force was kept constant. Then, the force was removed (ɛ_fix_) and sample was again heated to 37 °C with a heating rate of 2.5 °C min^−1^ and then held isothermally for 6 min (ɛ_end_). The shape fixity (*R*_f_) and shape recovery ratio (*R*_r_) were calculated based on the strain (ɛ) at different points, as stated above, using Equations (5) and (6), respectively

(5) $$R_{f}=\;\frac{\varepsilon_{\mathrm{fix}}}{\varepsilon_{\max}}\;\times \;100$$

(6) $$R_{r}=\;\frac{\varepsilon_{\max}-\varepsilon_{\mathrm{end}}}{\varepsilon_{\max}}\;\times \;100$$

### Ex Vivo

Porcine small intestine was obtained from a local slaughterhouse (Zurich, Switzerland) and kept at −20 °C. The radiography was performed on a New Zealand white rabbit's cadaver obtained in the context of the protocol approved by the Zürich Cantonal Animal Ethics Committee (ZH069/18) and the experiments performed according to the Swiss Animal Welfare Act (TSchG, 455).

### Thermal Imaging

Thermal images and videos were obtained with CompactXR infrared camera (SeeK Thermal).

### Statistical Analysis

Statistical significance in cytotoxicity experiments between the treated groups and a negative control, as well as in the photothermal performance experiments between different treatments and a negative control was assessed by one‐way ANOVA with Tukey's comparison test with *p*‐value < 0.05 considered significant using OriginPro 2019 (OriginLab Corporation).

## Conflict of Interest

The authors declare no conflict of interest.

## Supporting information

Supporting Information

Supplemental Video 1

Supplemental Video 2

Supplemental Video 3

Supplemental Video 4

Supporting Information

Supporting Information

## Data Availability

The data that support the findings of this study are available in the supplementary material of this article.;
