# Supplementary material for: Digital Light 3D Printed Bioresorbable and NIR‐Responsive Devices with Photothermal and Shape‐Memory Functions
Source: Adv Sci (Weinh). 2022 Jul 27;9(27):2200907. doi: 10.1002/advs.202200907 (PMC9507367; doi:10.1002/advs.202200907)
Supplement: Supplementary file 1 — Supporting Information [file ADVS-9-2200907-s003.pdf]

ADVANCED SCIENCE
Open Access

Supporting Information

for *Adv. Sci.*, DOI 10.1002/advs.202200907

Digital Light 3D Printed Bioresorbable and NIR-Responsive Devices with Photothermal and Shape-Memory Functions

Nevena Paunović, Jessica Marbach, Yinyin Bao, Valentine Berger, Karina Klein, Sarah Schleich, Fergal Brian Coulter, Nicole Kleger, André R. Studart, Daniel Franzen, Zhi Luo and Jean-Christophe Leroux**

Supporting Information for

Digital Light 3D-Printed Bioresorbable and NIR-Responsive Devices with Photothermal and Shape-Memory Functions

Nevena Paunović, Jessica Marbach, Yinyin Bao, Valentine Berger, Karina Klein, Sarah Schleich, Fergal Brian Coulter, Nicole Kleger, André R. Studart, Daniel Franzen, Zhi Luo, Jean-Christophe Leroux**

The PDF file includes:

- Figure S1. Synthetic reaction scheme of poly(DLLA-*co*-CL) methacrylate.
- Figure S2. Optimization of synthetic protocol for AuNRs.
- Figure S3. Synthetic route of PEGylated AuNRs with UV-vis spectra corresponding to the product after each step.
- Figure S4. Photographs of AuNRs in NVP.
- Figure S5. Viscosity of 9k-photopolymer alone and its resins with and without AuNRs at different temperatures.
- Figure S6. DSC heating curves of 9k-photopolymer and DLP 3D printed composite material with 0.1 wt% AuNRs.
- Figure S7. Photothermal performance of 3D printed composites with various concentrations of AuNRs.
- Figure S8. Mechanical performance of 3D printed composites with various concentrations of AuNRs.
- Figure S9. Photothermal performance of the composite material with 0.1 wt% of AuNRs and the control without AuNRs upon exposure to a 808-nm laser.
- Figure S10. Thermal curve of water with composite-based DLP 3D printed cuboid exposed for 15 min to 808-nm laser.
- Figure S11. The radiograph of a composite stent with 0.1 wt% AuNRs positioned beneath the rabbit's cadaver.
- Figure S12. Compression curves of stents in wet and dry states incubated in MOPS buffer pH 7.4 at 37 °C at various time points.
- Figure S13. EDX images of gold on dried cuboids (4 × 3 × 1 mm) over 5 weeks of degradation in MOPS buffer pH 7.4 at 50 °C.
- Figure S14. Release of gold from a representative stent over 4 weeks of accelerated degradation.
- Figure S15. DSC heating curves of 15k-photopolymer and DLP 3D printed composite material with 0.1 wt% AuNRs.
- Figure S16. Synthetic reaction scheme of poly(DLLA-*co*-CL)-SH.
- Figure S17. UV-vis spectra of gold seeds, AuNRs, AuNRs functionalized with poly(DLLA-*co*-CL)-SH, and DLP printed shape-memory composite material with 0.1 wt% of AuNRs.
- Figure S18. Viscosity of shape-memory photopolymer alone and its resin with 0.1 wt% of AuNRs at different temperatures.
- Figure S19. Mechanical properties of shape-memory polymer-based materials.
- Figure S20. Resistance to creep.
- Figure S21. DMA of DLP printed shape-memory composites.
- Figure S22. Photothermal performance of shape-memory composite material with 0.1 wt% AuNRs over three consecutive cycles of NIR light irradiation.
- Figure S23. Evaluation of shape-memory properties of 15k-based composite with 0.1 wt% AuNRs.
- Figure S24. DMA of a shape-memory cycle of 15k-based DLP 3D printed composite with 0.1 wt% AuNRs.
- Figure S25. Shape recovery experiment with the control shape-memory meshed stent.
- Figure S26. *Ex vivo* setup for investigating shape recovery of shape-memory composite-based meshed stent.
- Figure S27. Mechanical performance of shape-memory composites at room temperature and 37 °C.

- Figure S28. ^{1}H NMR spectra of 4-arm poly(DLLA-*co*-CL)s.
- Figure S29. ^{1}H NMR spectra of 4-arm poly(DLLA-*co*-CL) methacrylates.
- Figure S30. ^{1}H NMR spectra of poly(DLLA-*co*-CL) disulfide and poly(DLLA-*co*-CL)-SH.

- Table S1. Characterization of 4-arm copolymers used for 3D printing before methacrylation.
- Table S2. Characterization of poly(DLLA-*co*-CL) disulfide and poly(DLLA-*co*-CL)-SH.

- Video S1. *Ex vivo* shape recovery of a stent in the porcine intestinal segment upon NIR light irradiation.

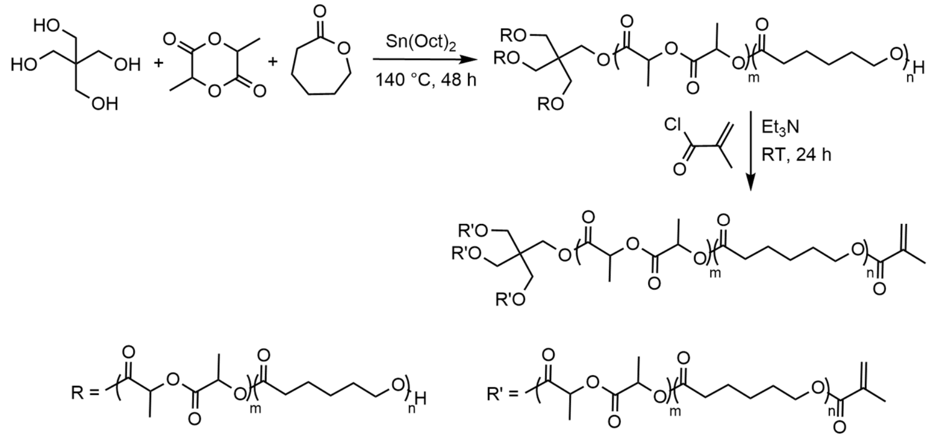

Figure S1. Synthetic reaction scheme of poly(DLLA-*co*-CL) methacrylate. Pentaerythritol was used as the initiator, m/n = 1/1, $M_{n\ NMR}$ = 8700 g mol^{-1} for 9k-polymer or m/n = 1/9, $M_{n\ NMR}$ = 15,200 g mol^{-1} for 15k-polymer.

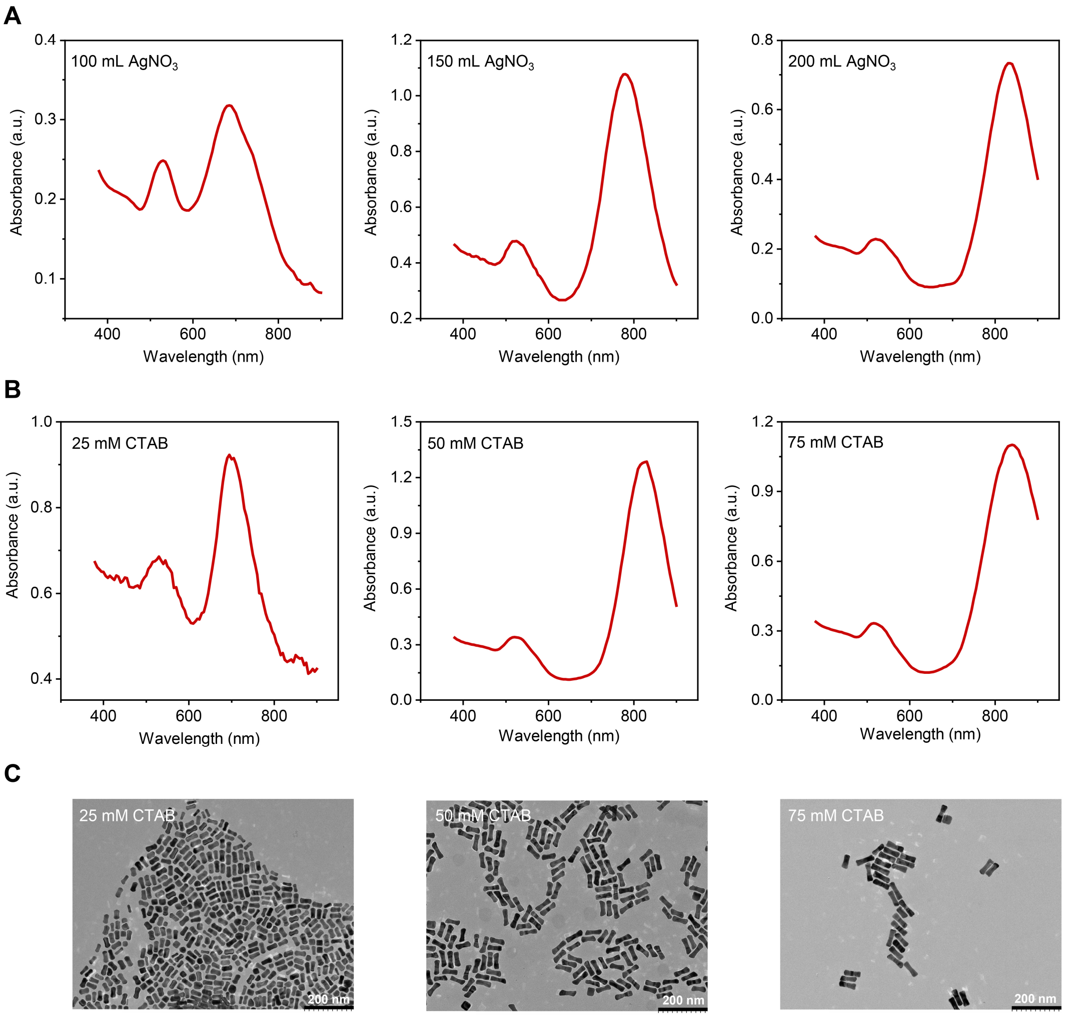

Figure S2. Optimization of synthetic protocol for AuNRs. (**A** and **B**) UV-vis spectra of AuNRs prepared with (**A**) varying amount of 4 mM silver nitrate ($AgNO_3$) and 182.2 mg of hexadecyl trimethylammonium bromide (CTAB) or (**B**) varying concentration of CTAB and 200 µL of 4 mM $AgNO_3$. (**C**) TEM images of AuNRs prepared with different CTAB concentrations. Average lengths of AuNRs with 25, 50 and 75 mM of CTAB were 43.0 ± 4.5 nm, 61.4 ± 6.0 nm, and 73.9 ± 6.1 nm, respectively (n = 30).

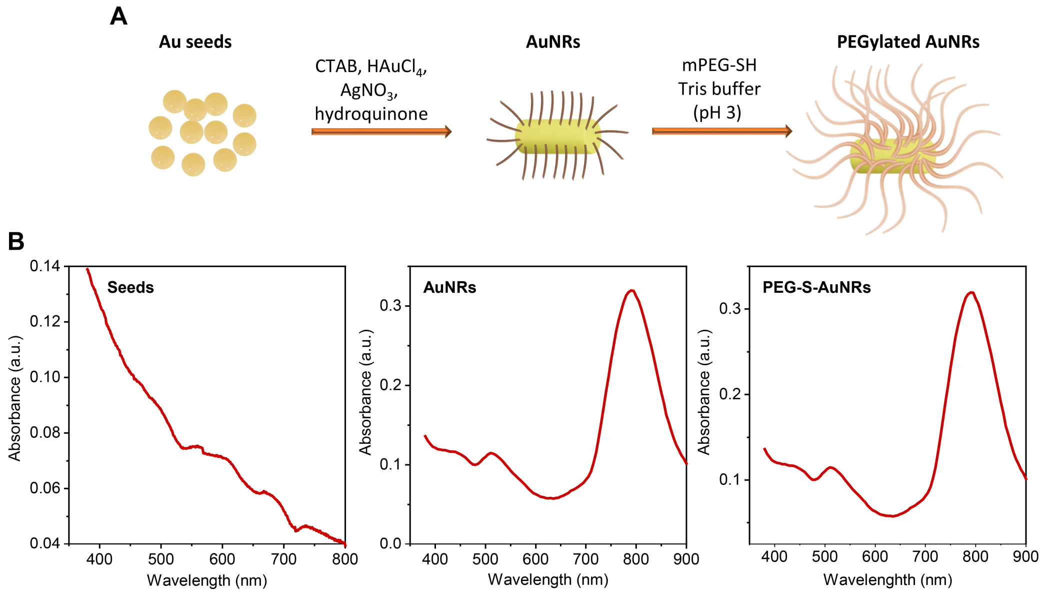

Figure S3. Synthetic route of PEGylated AuNRs with UV-vis spectra corresponding to the product after each step. (**A**) Schematic representation of the synthesis steps. (**B**) From left to right: gold seeds, AuNRs and PEGylated AuNRs. PEGylated AuNRs spectrum is the same as in Figure 2C and is presented here for clarity.

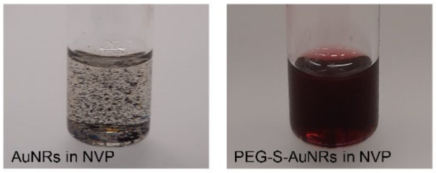

Figure S4. Photographs of AuNRs in NVP. Aggregated AuNRs before functionalization (left) and dispersed PEG-S-AuNRs (right) in NVP.

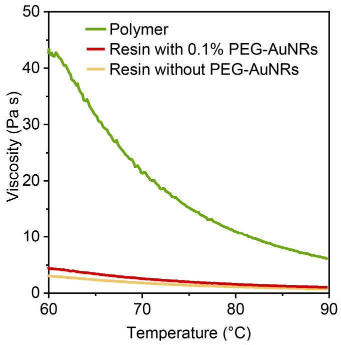

Figure S5. Viscosity of 9k-photopolymer alone and its resins with and without AuNRs at different temperatures. Viscosity of the resins at 85 °C (most commonly used printing temperature) was *ca.* 1.3 Pa s. 9k-photopolymer refers to poly(DLLA-*co*-CL) methacrylate, m/n = 1/1, $M_{n\ NMR}$ = 8700 g mol^{-1}.

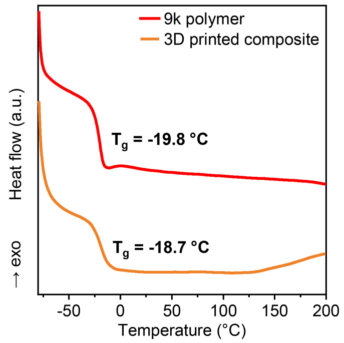

Figure S6. DSC heating curves of 9k-photopolymer and DLP 3D printed composite material with 0.1 wt% AuNRs. The tests were performed in heat-cool-heat mode and the second heating cycle is presented. Glass transition temperature (T_g) from this cycle is indicated on the graphs. The 9k-photopolymer refers to poly(DLLA-*co*-CL) methacrylate, m/n = 1/1, $M_{n\ NMR}$ = 8700 g mol^{-1}.

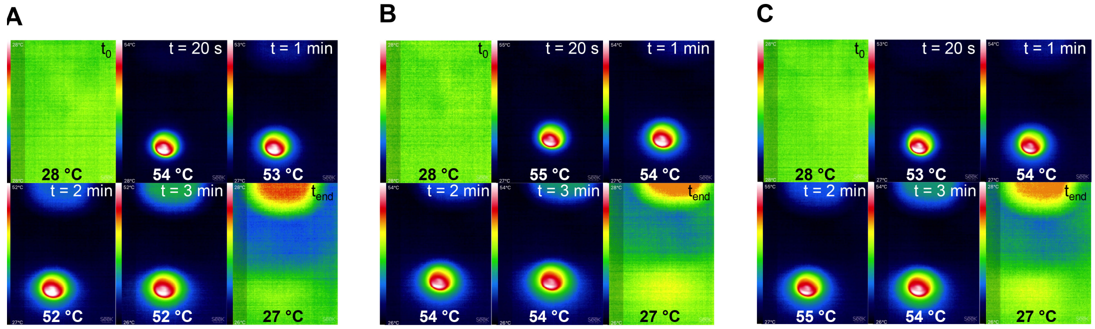

Figure S7. Photothermal performance of 3D printed composites with various concentrations of AuNRs. Thermal images of composite materials with (**A**) 0.07 wt%, (**B**) 0.13 wt%, and (**C**) 0.19 wt% of AuNRs at different time points over 3-min exposure to the laser light of 808 nm and 1 min after the irradiation was discontinued (t_{end}).

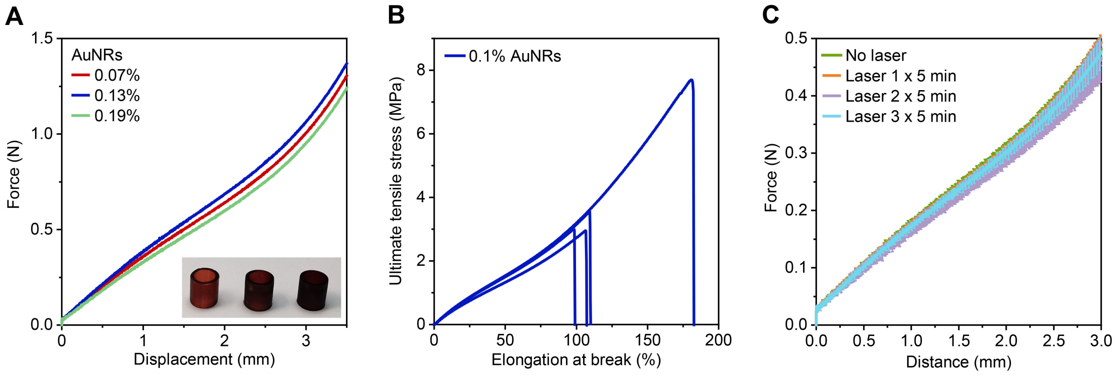

Figure S8. Mechanical performance of 3D printed composites with various concentrations of AuNRs. (**A**) Uniaxial compression force over distance average curves of DLP printed composites (H 10 mm, Ø 8 mm, thickness 1 mm). Each stent was compressed three times. Inset: Photograph of stents with 0.07, 0.13, and 0.19 wt% of AuNRs from left to right. (**B**) Engineering stress-strain curves of 3D printed composite containing 0.1 wt% of AuNRs. (**C**) Uniaxial compression curves of DLP printed composite stents with 0.1 wt% AuNRs (H 5 mm, Ø 8 mm, thickness 1 mm) before exposure to 808-nm laser and after one, two and three cycles of 5-min exposure. Mean ± s.d. (n = 3). Each stent was compressed three times.

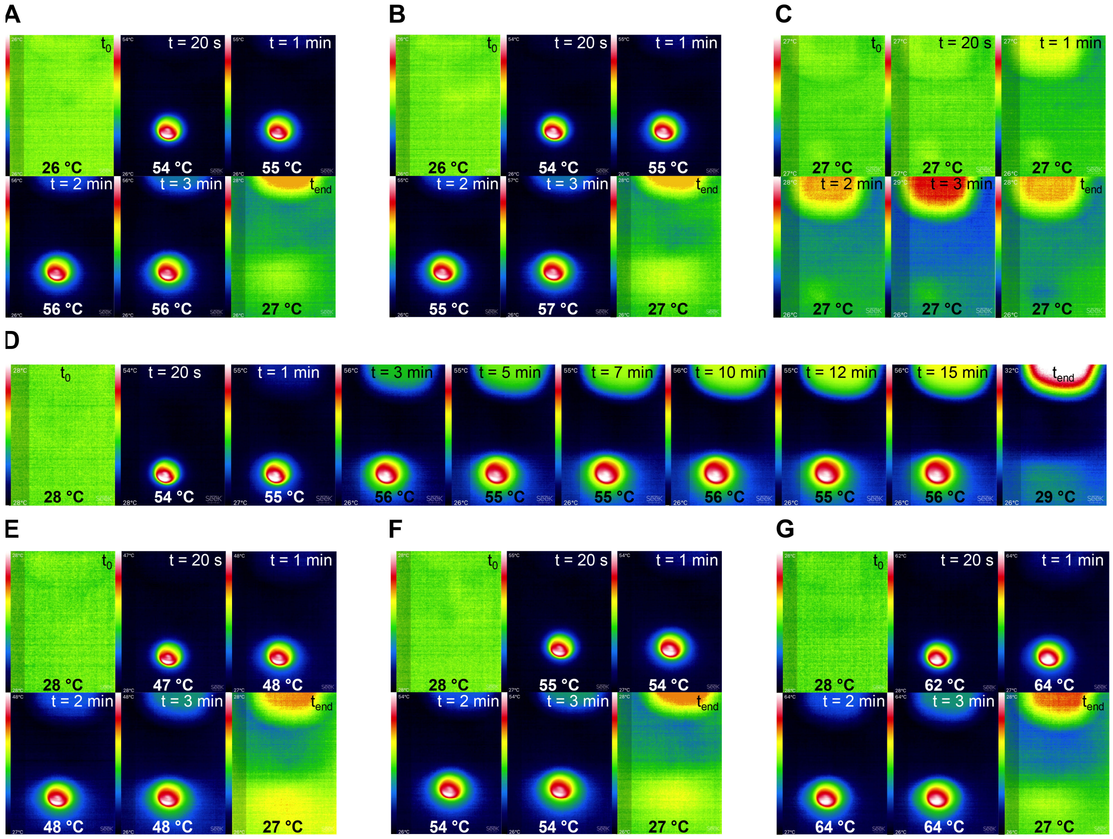

Figure S9. Photothermal performance of the composite material with 0.1 wt% of AuNRs and the control without AuNRs upon exposure to a 808-nm laser. (**A** and **B**) Thermal images of the composite material at different time points during (**A**) second and (**B**) third cycle of 3-min exposure. (**C**) Thermal images of the control material at different time points over 3-min exposure to the laser light. (**D**) Thermal images at various time points during continuous exposure of the composite material to the laser light over 15 min. (**E** to **G**) Thermal images over 3-min exposure of the composite materials of (**E**) 0.6 mm, (**F**) 0.8 mm, and (**G**) 1.0 mm thicknesses. Image corresponding to the end point of the experiments (t_{end}) was taken 1 min after the irradiation was stopped. Figure S7F is taken from Figure S5 and is presented here for comparison.

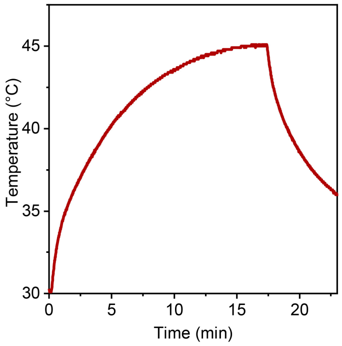

Figure S10. Thermal curve of water with composite-based DLP 3D printed cuboid exposed for 15 min to 808-nm laser. The experiment was performed at 37 °C with a 9k-based composite cuboid with 0.1 wt% AuNRs (13 × 7 × 0.8 mm).

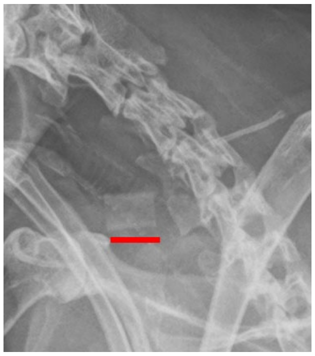

Figure S11. The radiograph of a composite stent with 0.1 wt% AuNRs positioned beneath the rabbit's cadaver. Position of the stent is marked with a red line.

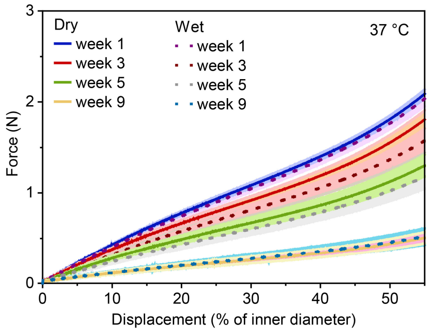

Figure S12. Compression curves of stents in wet and dry states incubated in MOPS buffer pH 7.4 at 37 °C at various time points.

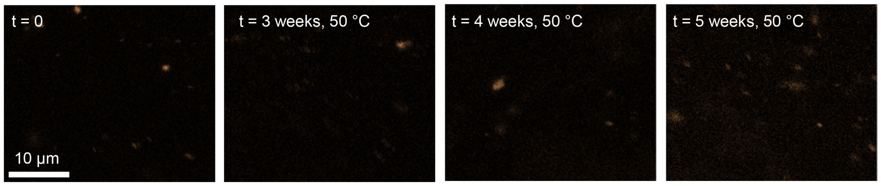

Figure S13. EDX images of gold on dried cuboids (4 × 3 × 1 mm) over 5 weeks of degradation in MOPS buffer pH 7.4 at 50 °C.

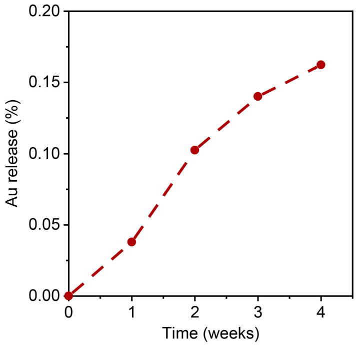

Figure S14. Release of gold from a representative stent over 4 weeks of accelerated degradation. Concentration of gold was determined by ICP-MS.

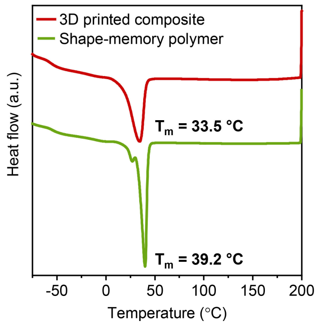

Figure S15. DSC heating curves of 15k-photopolymer and DLP 3D printed composite material with 0.1 wt% AuNRs. The tests were performed in heat-cool-heat mode and the second heating cycle is presented. Melting point (T_m) from this cycle is indicated on the graphs, while glass transition temperature is -55.6 °C and -58.7 °C for shape-memory polymer and 3D printed composite, respectively. The 15k-photopolymer refers to poly(DLLA-*co*-CL) methacrylate, m/n = 1/9, $M_{n\ NMR}$ = 15,200 g mol^{-1}.

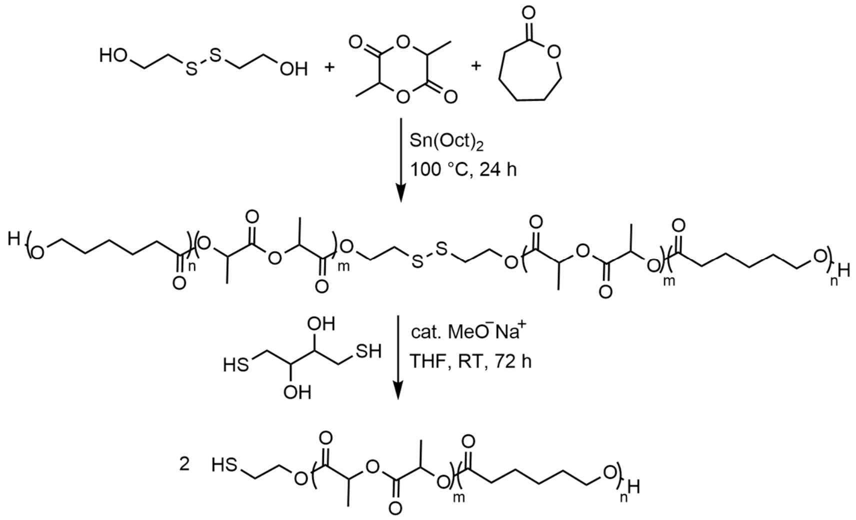

Figure S16. Synthetic reaction scheme of poly(DLLA-*co*-CL)-SH. The ligand was synthesized with LA to CL molar ratio (m to n) of 1 to 9 with $M_{n\ NMR}$ of approx. 4500 g mol^{-1}.

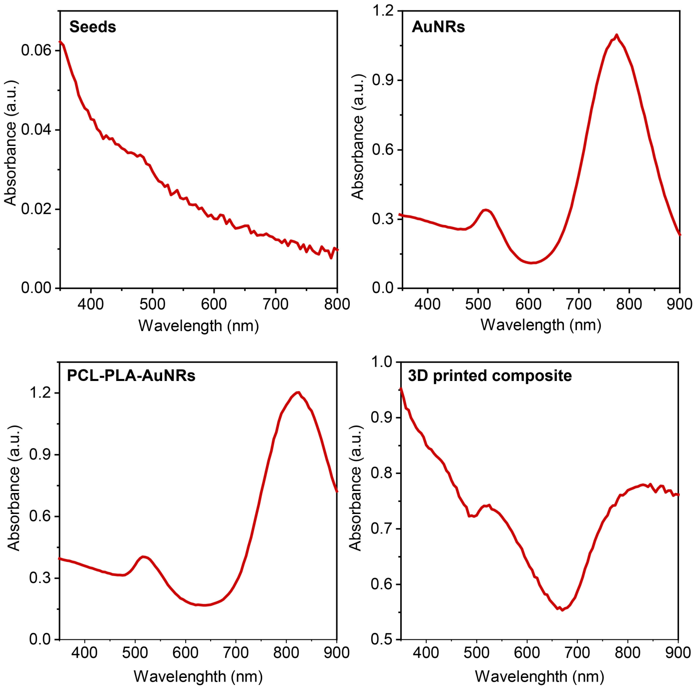

Figure S17. UV-vis spectra of gold seeds, AuNRs, AuNRs functionalized with poly(DLLA-*co*-CL)-SH, and DLP printed shape-memory composite material with 0.1 wt% of AuNRs.

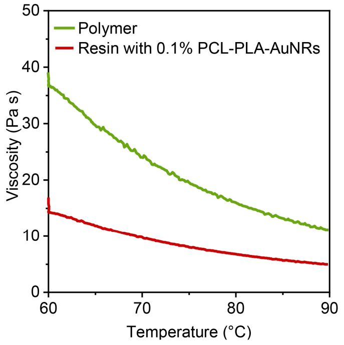

Figure S18. Viscosity of shape-memory photopolymer alone and its resin with 0.1 wt% of AuNRs at different temperatures. Viscosity of the resin at 85 °C (most commonly used printing temperature) was 5.7 Pa s.

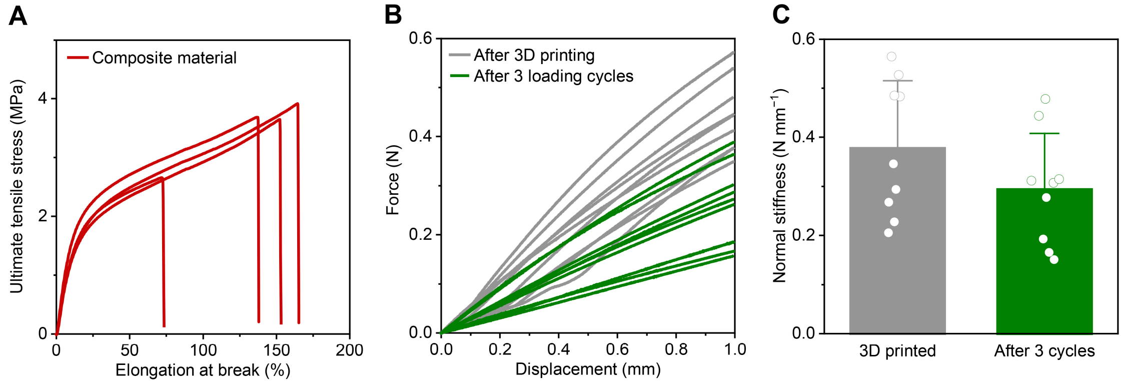

Figure S19. Mechanical properties of shape-memory polymer-based materials. (**A**) Engineering stress-strain curves of composite material before the programming step. (**B**) Uniaxial compression curves of three stents (H 4.6 mm, Ø 7.2 mm, thickness 0.85 mm) obtained before and after 3 consecutive loading cycles (deformation at 80 °C, preservation of the shape at -20 °C for 10 min and recovery over 1-min 808-nm laser exposure). (**C**) Normal stiffness calculated as a slope of the force-displacement curves (first 100 points). Mean + s.d. (n = 9, three stents compressed three times).

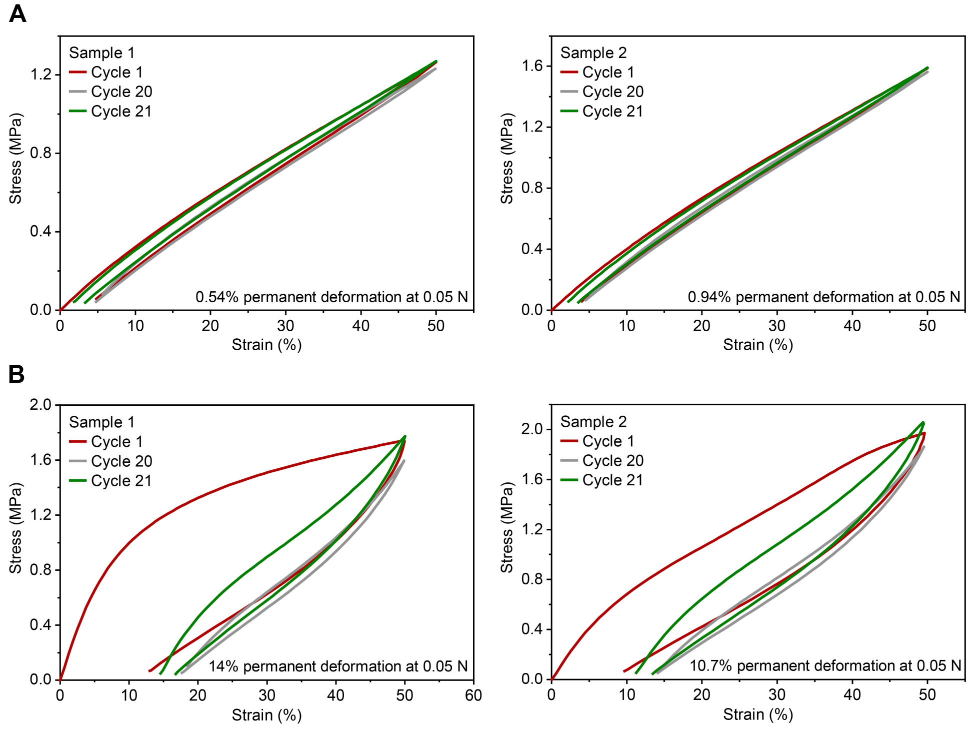

Figure S20. Resistance to creep. (**A** and **B**) Cyclic deformation experiments with two independent specimens of 9k- (**A**) and 15k-composite (**B**). The dog bone-shaped specimens (n =2) were deformed over 20 cycles to 50% of their length and the permanent deformation (indicated on the graphs) was determined after 2 h of recovery at the beginning of the 21st cycle at a force of 0.05 N.

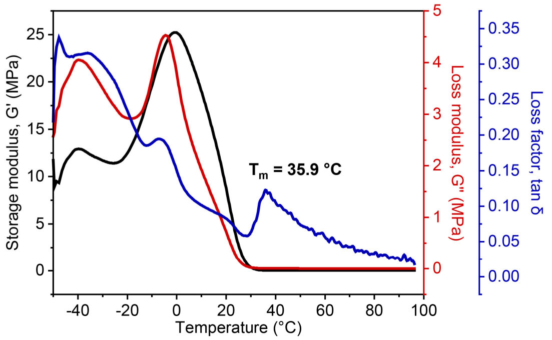

Figure S21. DMA of DLP printed shape-memory composites. Storage modulus (G'), loss modulus (G'') and loss factor (tan δ) as functions of temperature.

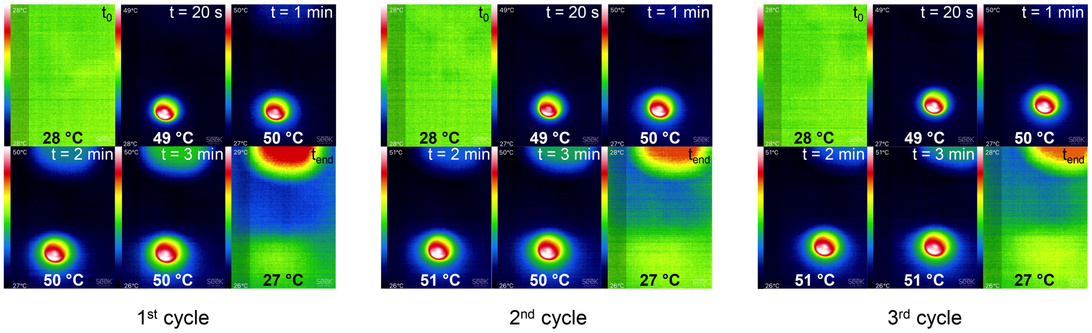

Figure S22. Photothermal performance of shape-memory composite material with 0.1 wt% AuNRs over three consecutive cycles of NIR light irradiation. Thermal images at different time points over 3-min exposure to the laser light (808 nm) and 1 min after irradiation was discontinued (t_{end}) obtained in three consecutive cycles.

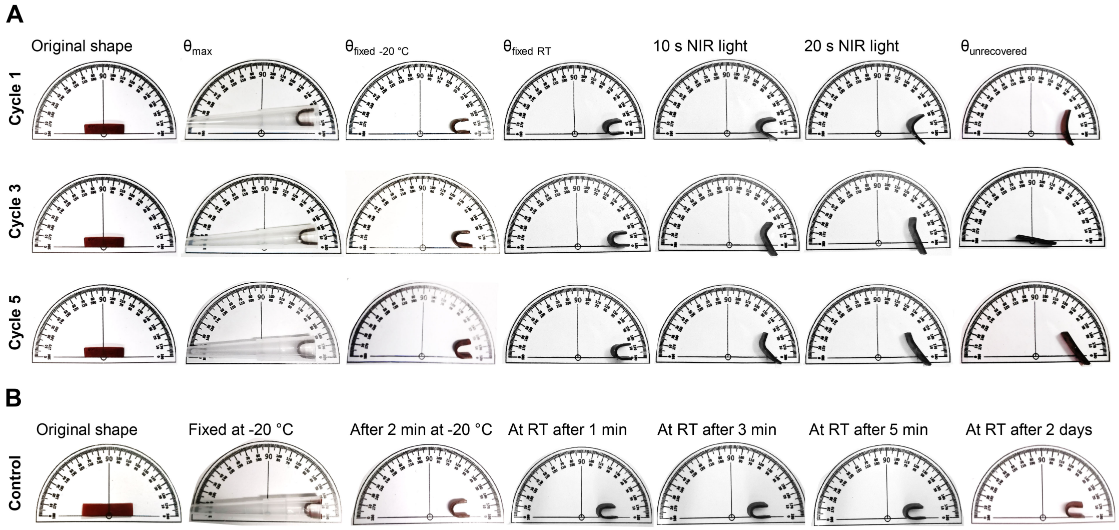

Figure S23. Evaluation of shape-memory properties of 15k-based composite with 0.1 wt% AuNRs. (**A**) Photographs of cuboids from original to deformed shape preserved at -20 °C (θ_{max}), after removal of external force at -20 °C for 2 min ($\theta_{fixed\ -20\ °C}$), after additional 2 min at room temperature ($\theta_{fixed\ RT}$) and after 1 min of exposure to 808-nm laser ($\theta_{unrecovered}$). Experiments were performed with three specimens (12.5 × 2.4 × 0.8 mm) over 5 cycles. (**B**) Photographs of a control cuboid from original to deformed shape preserved at -20 °C, after removal of external force at -20 °C for 2 min, after additional 5 min and after 2 days at room temperature.

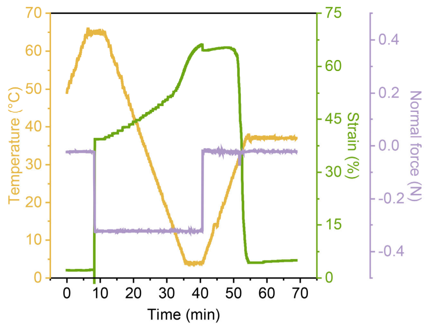

Figure S24. DMA of a shape-memory cycle of 15k-based DLP 3D printed composite with 0.1 wt% AuNRs. Shape fixity rate at 4 °C was calculated to be 97.1%, while shape recovery rate at 37 °C was 92.5%.

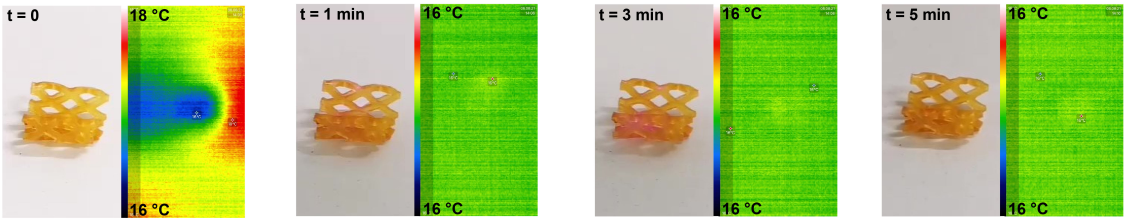

Figure S25. Shape recovery experiment with the control shape-memory meshed stent. Optical and thermal images of the meshed stent (H 11 mm, Ø 10.8 mm, thickness 1.1 mm) 3D printed with shape-memory polymer without AuNRs after the heating, folding, and freezing steps, upon the irradiation (808 nm) at different time points. The experiment was performed at *ca.* 16 °C.

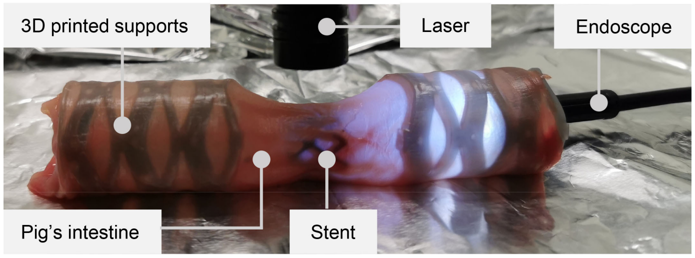

Figure S26. ***Ex vivo*** **setup for investigating shape recovery of shape-memory composite-based meshed stent.**

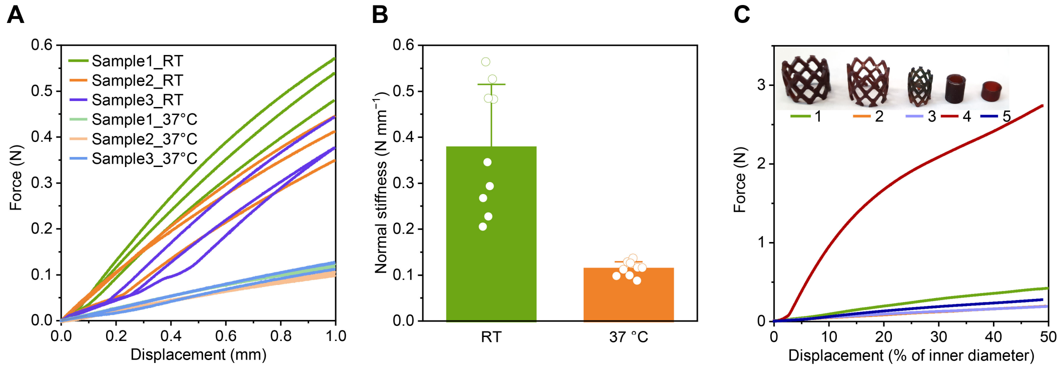

Figure S27. Mechanical performance of shape-memory composites at room temperature and 37 °C. (**A**) Uniaxial compression curves of 15k-based shape-memory composite stents (Ø 7.2 mm, H 4.6 mm, thickness 0.85 mm). One colour represents one sample compressed three times. (**B**) Normal stiffness calculated as a slope of the force-displacement curves (first 100 points). Mean + s.d. (n = 9, three stents compressed three times). (**C**) Force-displacement curves from uniaxial compression tests performed at 37 °C with five stents differing in size, thickness and design. Inset: Photograph of the tested stents. Dimensions of the stents: 1 (Ø 15.0 mm, H 12.9 mm, thickness 1.49 mm), 2 (Ø 14.5 mm, H 13.4 mm, thickness 1.20 mm), 3 (Ø 9.4 mm, H 11.2 mm, thickness 0.98 mm), 4 (Ø 7.1 mm, H 9.2 mm, thickness 0.90 mm) and 5 (Ø 7.2 mm, H 4.6 mm, thickness 0.85 mm). All samples contained 0.1 wt% AuNRs. Room temperature samples from (A) and (B) are taken from Figure 19 (B and C, respectively, 3D printed samples), and are presented here for comparison.

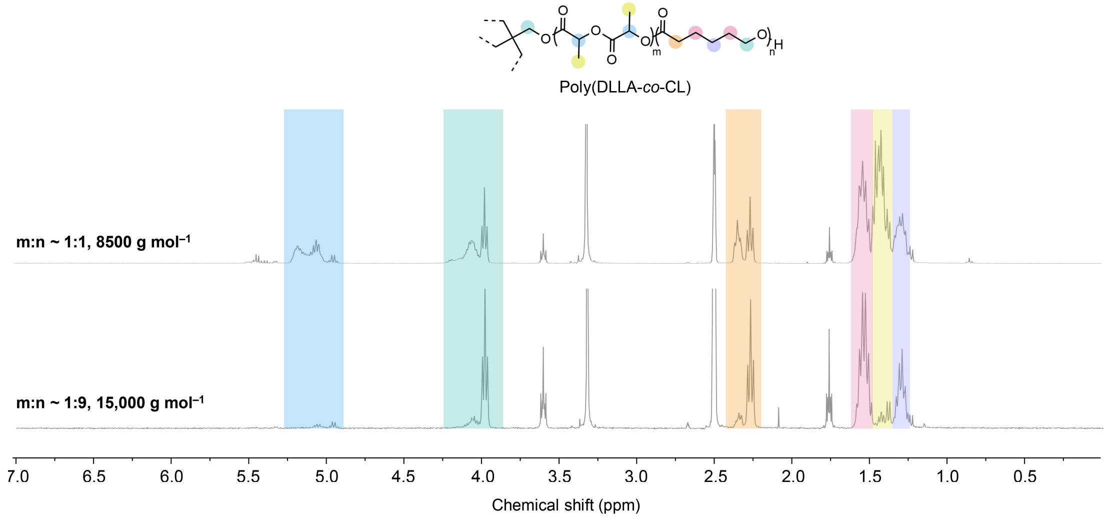

Figure S28. ^{1}H NMR spectra of 4-arm poly(DLLA-*co*-CL)s. Solvent: DMSO-d6. Solvent peaks appeared at 2.50 ppm (DMSO), 3.33 ppm (H_2O), 3.60 and 1.76 ppm (THF), and 1.25 and 0.86 ppm (*n*-hexane). The signals of unreacted LA and CL are located at 5.3-5.5 ppm and 2.7 ppm, respectively.

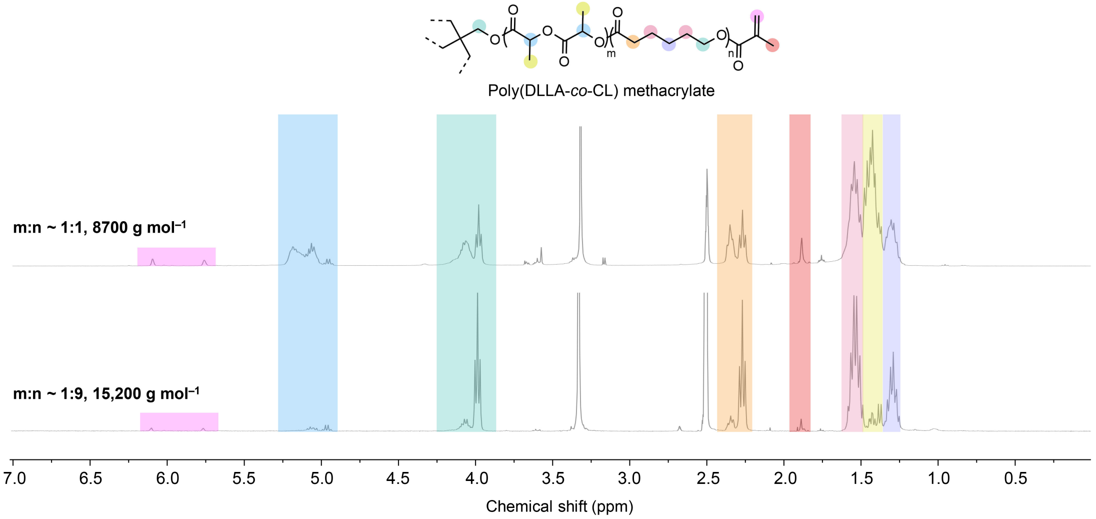

Figure S29. ^{1}H NMR spectra of 4-arm poly(DLLA-*co*-CL) methacrylates. Solvent: DMSO-d6. Solvent peaks appeared at 2.50 ppm (DMSO), 3.33 ppm (H_2O), 3.60 and 1.76 ppm (THF), and 3.16 ppm (methanol).

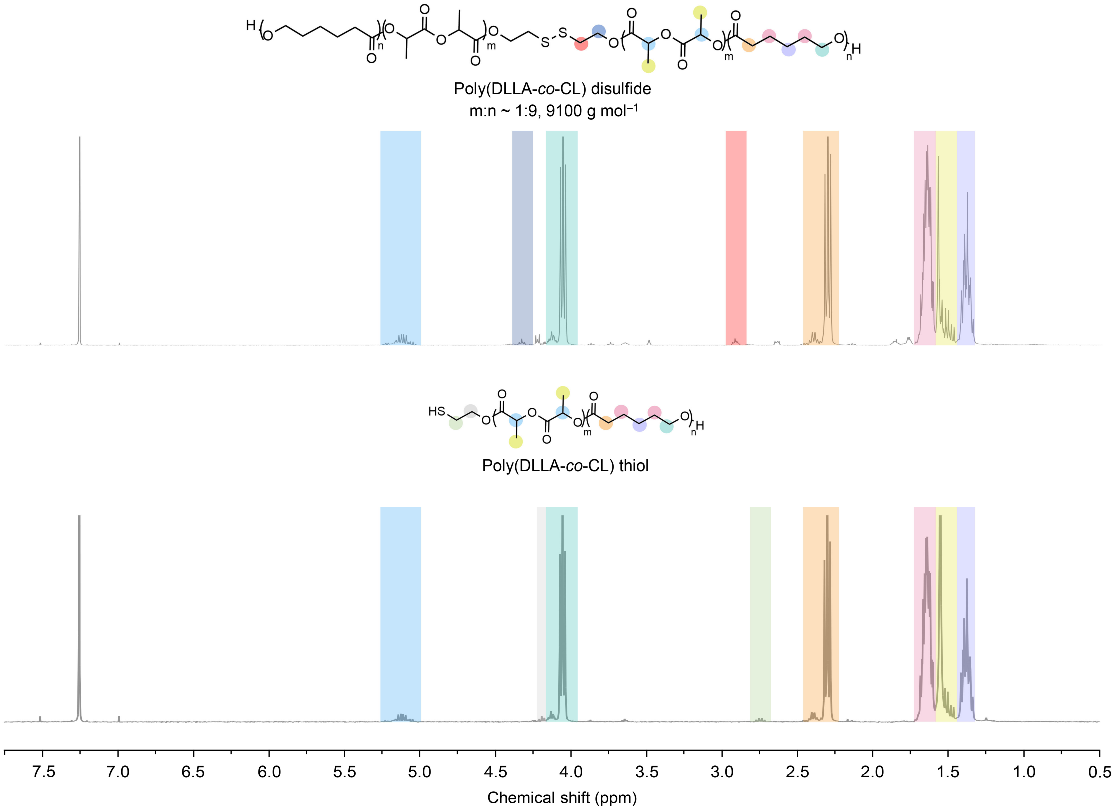

Figure S30. ^{1}H NMR spectra of poly(DLLA-*co*-CL) disulfide and poly(DLLA-*co*-CL)-SH. Solvent: $CDCl_3$. Solvent peaks appeared at 7.26 ppm ($CDCl_3$), 3.76 and 1.85 ppm (THF), and 3.49 and 1.09 ppm (methanol).

Table S1. Characterization of 4-arm copolymers used for 3D printing before methacrylation.[a)]

Polymer	$M_{n\ NMR}$[b)] (g mol^{-1})	[CL]/[LA][c)] (mol/mol)	$M_{n\ SEC}$[d)] (g mol^{-1})	Đ[d)]
9k	8500	34/31	15,400	1.24
15k	15,000	115/12	27,200	1.25

a) Unfunctionalized polymers were used for SEC experiments to prevent potential crosslinking on the columns.

b) Molecular weight of unfuctionalized polymer calculated from NMR spectra based on the conversion of LA ($LA_{\%}$) and CL ($CL_{\%}$) using Eq. 1, 2, and 3

$$LA_{\%} = \frac{A_{5.2\ ppm}}{A_{5.2\ ppm} + A_{5.4\ ppm}} \times 100 \quad (1)$$

$$CL_{\%} = \frac{A_{2.3\ ppm}}{A_{2.3\ ppm} + A_{2.7\ ppm}} \times 100 \quad (2)$$

$$M_n = MW_{initiator} + \frac{N_{LA} \times DLLA_{\%}}{100} \times MW_{LA} + \frac{N_{CL} \times CL_{\%}}{100} \times MW_{CL} \quad (3)$$

where A is the peak integral at a specific chemical shift, and N is the number of equivalents of a monomer used in the synthesis.[1]

c) Degree of polymerization calculated from NMR spectra based on comparison of equivalents of the monomers in the polymer obtained by multiplying conversion of the monomers ($LA_{\%}$ and $CL_{\%}$) with corresponding initial number of equivalents (N_{LA} and N_{CL}).[1]

d) Number average molecular weight (M_n) and polydispersity index (Đ) calculated from SEC spectra.

Table S2. Characterization of poly(DLLA-*co*-CL) disulfide and poly(DLLA-*co*-CL)-SH.

Polymer	$M_{n\ NMR}$[a)] (g mol^{-1})	[CL]/[LA][a)] (mol/mol)	$M_{n\ SEC}$[b)] (g mol^{-1})	Đ[b)]
poly(DLLA-*co*-CL) disulfide	9100	8/68	17,400	1.29
poly(DLLA-*co*-CL)-SH	n.a.	n.a	8,100	1.73

a) Molecular weight and degree of polymerization were calculated based on DMSO-d6 spectra as described in Table S1.

d) Number average molecular weight (M_n) and polydispersity index (Đ) calculated from SEC spectra.

n.a.: not applicable

References

[1] N. Paunović, Y. Bao, F. B. Coulter, K. Masania, A. Karoline, K. Klein, A. Rafsanjani, J. Cadalbert, P. W. Kronen, A. Karol, Z. Luo, F. Rüber, D. Brambilla, B. Von, D. Franzen, A. R. Studart, J.-C. Leroux, *Sci. Adv.* **2021**, *7*, abe9499.
